# Replication-transcription collisions impose DNA strand-specific constraints on gene length in bacteria

**DOI:** 10.1371/journal.pgen.1012282

**Published:** 2026-08-24

**Authors:** Anjali Variyar, Samhitha Patil, Jebin Babu, Akanksha Bhat, T. Sabari Sankar

**Affiliations:** 1 School of Biology, Indian Institute of Science Education and Research, Thiruvananthapuram, Kerala, India; 2 Center for High-Performance Computing, Indian Institute of Science Education and Research, Thiruvananthapuram, Kerala, India; University of Wisconsin-Madison, UNITED STATES OF AMERICA

## Abstract

Gene length is a peculiar genomic feature that exhibits minimal variation within domains of life, suggesting universal underlying constraints. The length of a gene is primarily influenced by its function, expression level, and mutation risk. Interestingly, many of these factors vary depending on whether the gene is located on the leading or lagging strand of DNA replication. Studies in bacterial species *E. coli* and *B. subtilis* have shown that genes tend to be shorter on the lagging strand, potentially reflecting selection to minimize head-on encounters between the replication and transcription machinery. However, the universality of strand-specific constraints on gene length and the evolutionary basis remain unexplored. Here, using comparative genomics, we analyzed gene lengths in the bacterial domain and revealed a non-neutral distribution of gene lengths across leading and lagging strands. Genes and operons are consistently shorter on the lagging strand, irrespective of essentiality or functionality. The length restriction was more pronounced in bacterial species with a dual DNA polymerase mode of replication, which may experience severe head-on collisions between replication and transcription. Remarkably, we found that with increasing length of transcription units, substitution rates increased in the promoters on the lagging strand rather than the coding sequences, revealing a length-dependent and lagging strand-specific cis-regulatory mutational susceptibility. Together, we uncovered a pervasive selection pressure that optimizes gene lengths in a DNA strand-specific manner across bacteria to preserve the genetic integrity of promoters.

## Author summary

Biological novelty is directly influenced by gene length, which is linked to the function of the encoded protein, gene expression level, and the risk of accumulating mutations. Hence, gaining insights into the molecular and evolutionary factors that govern gene length is fundamental to understanding the evolution of biological function. Across domains of life, from bacteria to humans, gene lengths seem to be very restricted around a mean of approximately 1000–1500 nucleotides. Additionally, in certain bacterial species, mean gene lengths were observed to vary by DNA replication strand. In this study, we compared the gene length distribution of the entire bacterial kingdom and found that gene length is highly restricted, strikingly on a DNA strand-specific basis. We found that genes are generally shorter on the lagging strand and longer genes tend to accumulate mutations in their regulatory regions. We propose that the two fundamental processes of replication and transcription that traverse DNA, often leading to molecular conflicts, impose the strand-specific selection pressure limiting the gene length. Thus, minimizing the adverse consequences of replication-transcription conflicts seems to delimit gene lengths and the evolution of biological function.

## Introduction

Gene length is an elementary and highly conserved genomic feature with minimal variation within each domain of life, including bacteria. The narrow range of gene lengths in the bacterial domain contradicts the otherwise highly variable genomic features of bacteria, such as genome size, gene number, GC content, and gene-strand bias [1–4]. Gene length is known to be influenced by multiple interrelated factors, including the size and function of the encoded RNA or protein, and gene expression level, in addition to mutation and selection [4–6]. Generally, gene length increases with the functional complexity of the protein product, but longer genes and proteins incur higher energy costs [4,5]. Consequently, it was proposed that functional complexity is mostly achieved through an increase in non-coding DNA [7]. Further, gene length was found to be inversely proportional to gene expression level, and it has also been observed that the length of a protein-coding gene can be influenced by its interacting partners or associated subunits in a complex [8–10]. Finally, longer genes may suffer from the increased risk of mutations and pseudogenization [6,11]. Overall, gene length is proposed to be controlled to ensure functionality and higher transcriptional/translational efficiencies, while preserving genetic integrity. Intriguingly, most of these factors governing gene length are highly DNA strand-specific, with differences between the leading and lagging strands of replication.

The intrinsic strand-specificity of DNA replication results in differential interaction with transcription. Replication-transcription collisions (RTCs) happen inevitably when the replisome and transcription complex traverse the same DNA template simultaneously [12,13]. RTCs are of two types: co-directional, when replication and transcription machinery travel on the leading strand in the same direction, and head-on, when both machinery travel opposite to each other on the lagging strand. Both collisions cause replication and transcription stress, leading to genomic instability, with the effect being far more severe for head-on RTCs on the lagging strand [13,14]. In this context, an earlier study using a statistical physics model predicted that longer transcription units would be favored on the leading strand of replication to reduce the number of interrupted transcripts resulting from head-on encounters between the replication and transcription machinery [15]. Similarly, it has also been proposed that longer transcripts, as in operons, are preferentially encoded on the leading strand, presumably to avoid longer interruptions during transcription on the lagging strand [16]. Thus, it is possible that the physical encounters of the two fundamental processes on the DNA in a strand-specific manner could significantly influence the length of the gene. Such a strand-specific bias in gene length was previously observed in *B. subtilis*, where leading strand genes were on average 48% longer than genes on the lagging strand [17].

Overall, it is well accepted that gene length, consequently, protein length is under a strong evolutionary constraint across life forms [4,7]. However, the nature and mechanism underlying the selective constraints remain less understood. Here, we systematically analyzed the strand-specific distribution of gene lengths on the leading and lagging strands across the bacterial domain and found that gene length distributions differ significantly between the strands. Genes and operons are consistently shorter on the lagging strand. We found a strong association between replication machinery and RTCs with gene lengths, highlighting a mechanistic and evolutionary basis for DNA strand-specific constraint on gene length. Our analyses revealed a length-dependent and lagging strand-specific increase in the promoter mutation rate uncovering cis-regulatory elements as potential targets of selection for restricting gene length.

## Results

### DNA replication strand-specific distribution of gene lengths

**Fig 1:**
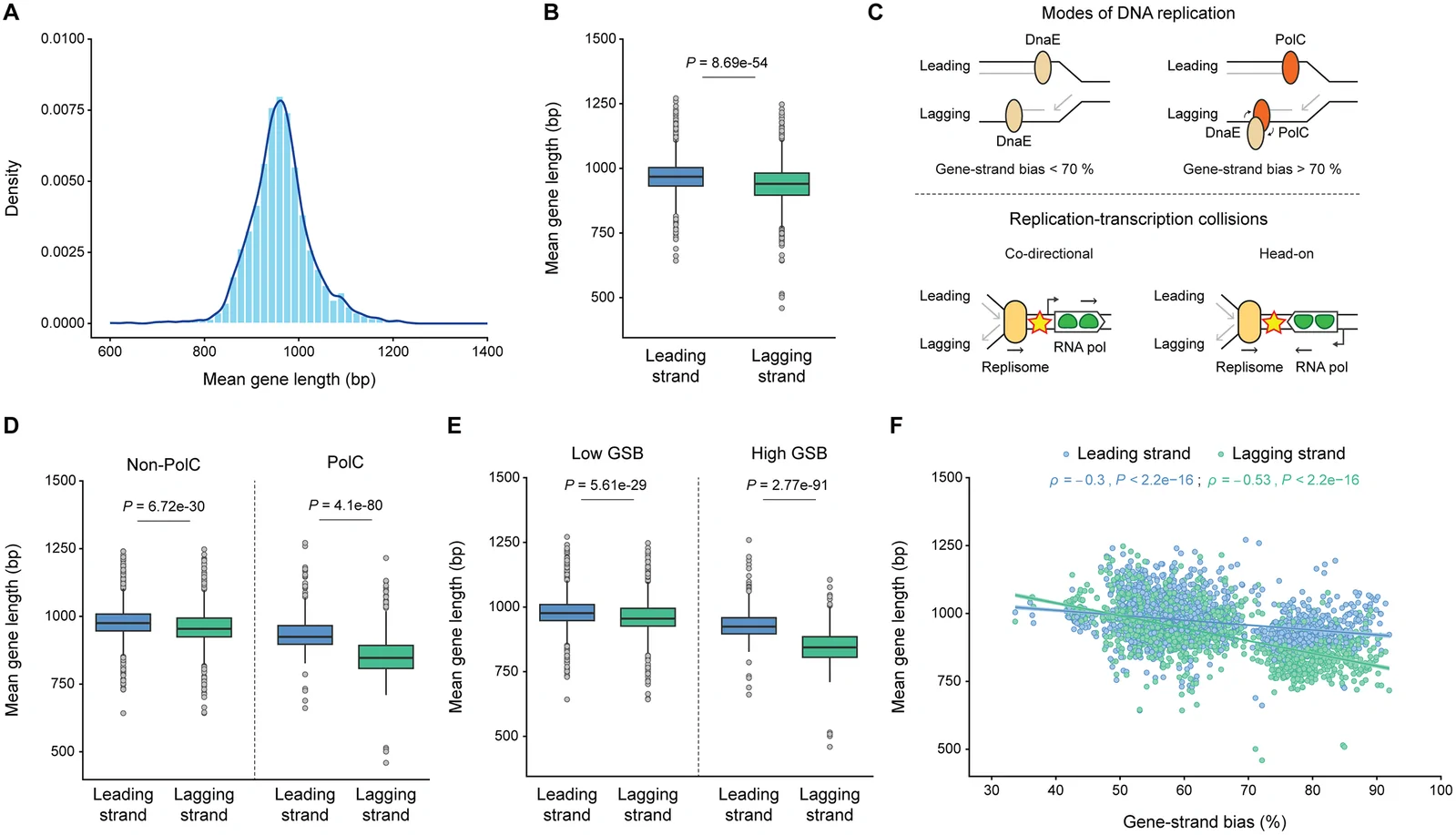
DNA strand-specific differences in gene length in bacteria. **(A)** Distribution of mean gene length of 2368 species spanning the bacterial domain. The histogram is plotted with a 50 bp bin size. **(B)** Distribution of mean length of genes on leading and lagging strands of DNA replication (2368 species). **(C)** Schematic of the two different modes of DNA replication and replication-transcription collisions in bacteria. **(D)** Distribution of mean gene lengths on leading and lagging strands in species lacking (non-PolC, DnaE present; n = 1851) or having PolC (DnaE and PolC present; n = 517). **(E)** Distribution of mean gene lengths on leading and lagging strands in low (n = 1842) and high (n = 526) GSB species. **(F)** Correlation between mean gene length and GSB. Linear regression fits for leading and lagging strand genes are presented with 95% confidence intervals (2368 species). Spearman’s rank correlation coefficients (*ρ*) and the *P*-values are presented on the plot. In **B**, **D**, and **E**, statistical significance was calculated using the Mann-Whitney *U*-test and *P*-values are indicated on the plot. Gene length is expressed in base pairs (bp). Number of species is indicated by n**.**

To examine whether the distribution of gene lengths is influenced by the strand of DNA replication in bacteria, we determined the mean gene lengths of 2,368 reference/representative species spanning the entire bacterial domain, encompassing nearly 30 phyla. We observed that the distribution of mean gene lengths of the bacterial domain was centered around 961 bp, aligning closely with the previous analyses [2–4] (Fig 1A). Further, ~ 74% of the species have mean gene lengths within 1 standard deviation (896–1027 bp), reaffirming the narrow distribution of gene lengths in bacteria. Strikingly, while the differences between the minima and maxima of genome size and number of genes were up to ~ 22-fold, the mean gene lengths varied only up to 2-fold. Although the number of genes increases in strong correlation with an increase in coding sequence length and thereby genome size, the mean gene length remains nearly constant (S1A Fig). Next, we divided genes into those encoded on the leading and lagging strands of DNA replication and analyzed their length distributions. We found that the distribution of lagging strand gene lengths had a significantly lower median than that of leading strand genes, indicating that genes are shorter on the lagging strand (Fig 1B). On average, leading strand genes were 972 bp long, whereas genes on the lagging strand were 936 bp in length, amounting to a ~ 4% difference.

Broadly, in bacteria, the mode of replication exhibits dichotomy [18]. Although the mechanism of bacterial replication is highly conserved, the replisome machinery is of dual nature (Fig 1C). Gram-negative bacteria such as *E. coli* use two or more copies of the same catalytic ⍺ subunit, DnaE, for both leading and lagging strand replication. On the other hand, Gram-positive species such as *B. subtilis* employ two different catalytic ⍺ subunits, DnaE and PolC, wherein PolC replicates the leading strand, while both PolC and DnaE are involved in lagging strand replication [18,19]. The distinct difference in the mode of replication is strongly associated with a bias in the distribution of genes between the DNA strands, referred to as gene-strand bias (GSB), which is calculated as the percentage of genes encoded on the leading strand [20–23]. Bacteria have evolved GSB to minimize the consequences of replication-transcription collisions (Fig 1C) [24,25]. Selection promotes GSB to co-orient replication and transcription since co-directional RTCs on the leading strand are relatively less detrimental than head-on RTCs on the lagging strand [25–27]. Therefore, we hypothesized that the distinct replication modes and differential fitness effects of RTCs could influence gene length in a strand-specific manner.

To examine the influence of the nature of replication and RTCs on gene length, we delineated species based on the replisome composition into non-PolC (only DnaE present) and PolC groups (both DnaE and PolC present) and those with low (< 70%) and high (> 70%) GSB, based on the reported bimodal distribution in bacteria [22,23]. In all the groups, the lagging strand genes were significantly shorter than those on the leading strand, and the difference was more pronounced in species possessing PolC and having high GSB (Fig 1D and 1E). While the difference in mean lengths between leading and lagging strands was modest, ~ 2% in the non-PolC/low GSB group (non-PolC – leading: 981 bp, lagging: 960 bp; low GSB – leading: 983 bp, lagging: 962 bp), it was prominent with a mean difference of ~10% in PolC/high GSB species (PolC – leading: 938 bp, lagging: 853 bp; high GSB – leading: 933 bp, lagging: 846 bp) (S1 and S2 Tables). Interestingly, the mean gene length in PolC and high GSB species was generally shorter on both strands than that in non-PolC and low GSB species, with notable mean differences of 107 bp and 117 bp, respectively, for lagging strand genes (Fig 1D and 1E; S1 and S2 Tables). Since the majority of species with high GSB possess PolC and the gene length distributions were almost identical across both classifications, we carried out most of the analyses employing GSB classification. In order to account for the species-specific differences, we compared the *z*-scores of gene lengths and found that consistently lagging strand genes are shorter, particularly in high GSB species (S1B and S1C Fig). We next examined the gene lengths by categorizing species into slow- and fast-growing to assess whether growth rate influences gene length. We found that lagging strand-specific constraints on gene length were evident in both the slow- and fast-growing species (S2A and S2B Fig). We further noted that ~94% of the analyzed species possess circular chromosomes and 6% of species have linear chromosomes, which also exhibited strand-specific variation in gene length (S3A and S3B Fig). In the high GSB group, lagging strand genes remain significantly shorter, while the low GSB group shows a modest increase in lagging strand gene length. This inconsistency could likely arise from the difficulty of accurately predicting the origin/terminus of linear chromosomes.

**Fig 2:**
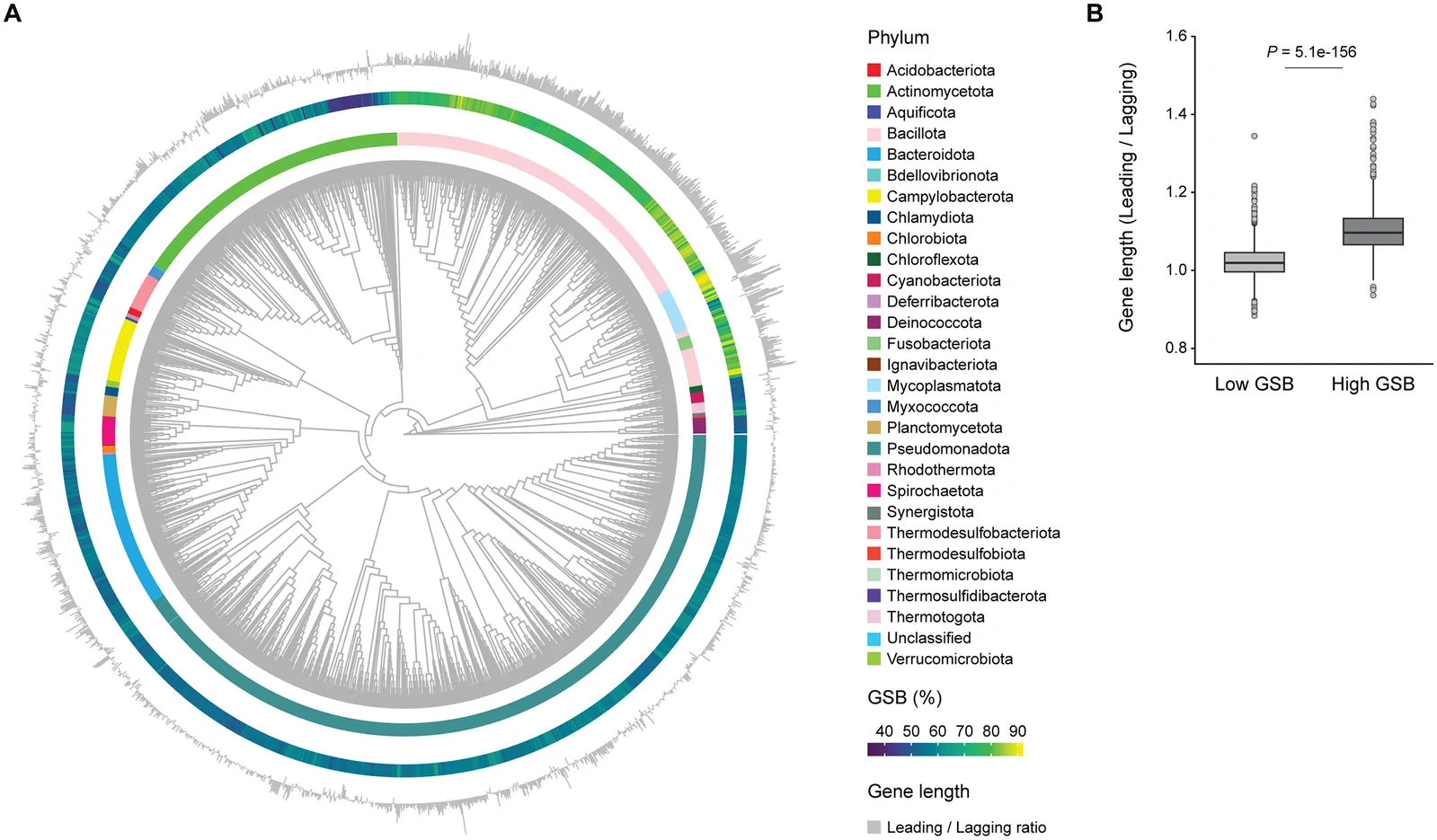
Replication-dependent distribution of gene lengths across bacterial domain. **(A)** Bacterial domain phylogeny depicting the bacterial taxa (1^st^ circle), GSB (2^nd^ circle), and ratio of mean gene lengths of leading over lagging strands presented as bars on a log scale (3^rd^ circle). The phylum information and the GSB scale are shown on the right. The height of the bars corresponds to the ratio. **(B)** Distribution of the ratio of mean lengths of genes on leading and lagging strands in low GSB (n = 1842) and high GSB (n = 526) species. Statistical significance was calculated using the Mann-Whitney *U*-test and *P*-value is indicated on the plot. Number of species is indicated by n**.**

Hence, these results suggest a strong link between replication and gene lengths, especially on the lagging strand and in species with PolC and high GSB. Further, to ascertain the same, we derived the correlation between the breadth of GSB and gene lengths. We found that the mean gene lengths on both the strands are negatively correlated with GSB with the effect being stronger for lagging strand genes (Fig 1F). The inverse relationship became apparent when we calculated the ratio of leading vs lagging strand gene lengths across the bacterial domain and mapped it onto the phylogenetic tree (Fig 2A and S4 Fig). An evident association was observed between GSB and gene length ratio across species, with greater ratios exhibited by high GSB species (Fig 2B), highlighting the replication-dependent selective constraint on gene lengths.

### Lagging strand genes are shorter irrespective of function

**Fig 3:**
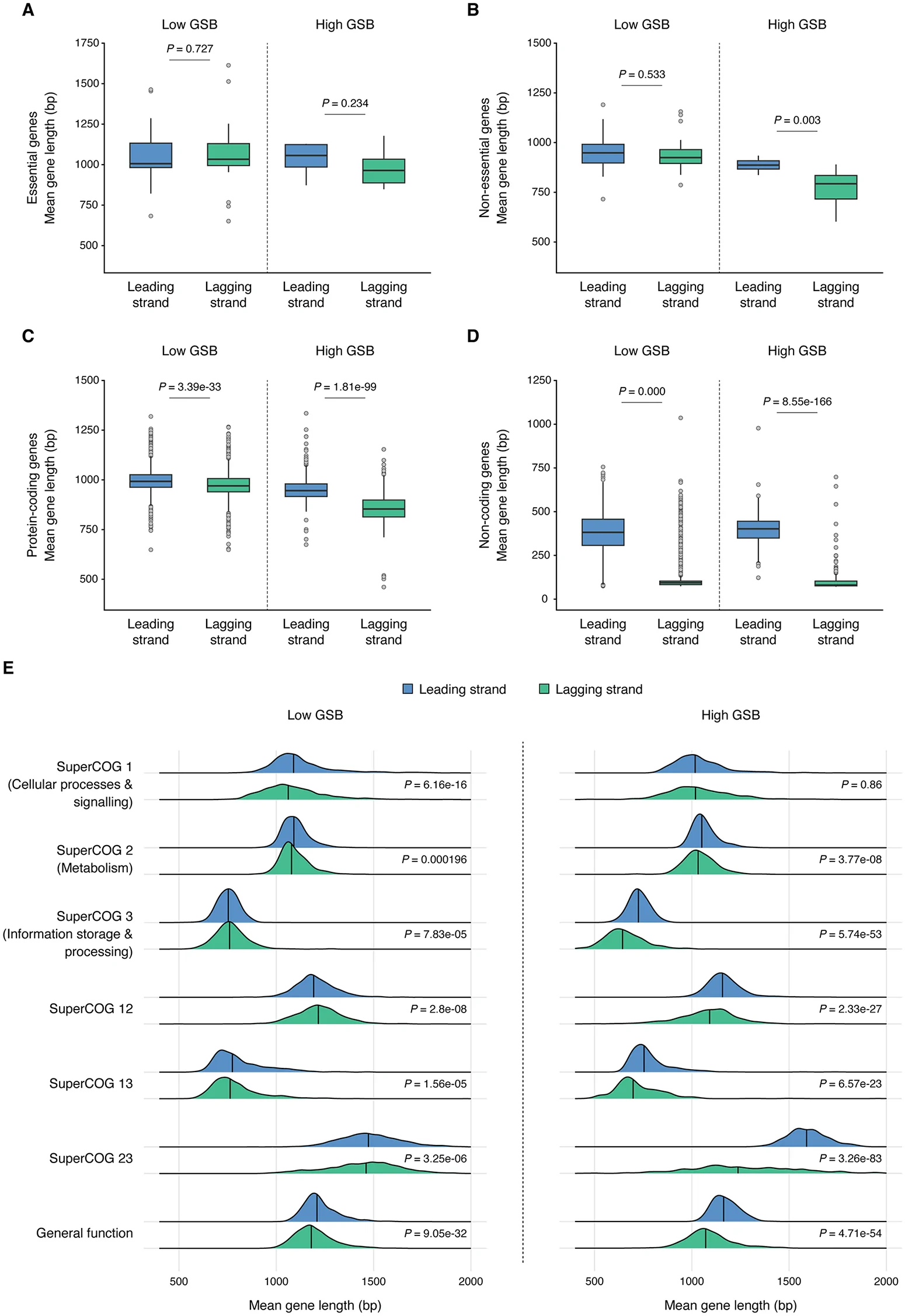
Genes are shorter on lagging strand regardless of functionality. **(A)** Distribution of mean length of essential genes on leading and lagging strands in low (n = 21) and high GSB (n = 8) species. **(B)** Distribution of mean length of non-essential genes on leading and lagging strands in low (n = 21) and high GSB (n = 8) species. **(C)** Distribution of mean length of protein-coding genes on leading and lagging strands in low (n = 1842) and high (n = 526) GSB species. **(D)** Distribution of mean length of non-coding genes on leading and lagging strands in low (leading, n = 1842; lagging, n = 1840) and high (leading, n = 526; lagging, n = 517) GSB species. **(E)** Distribution of mean gene lengths for SuperCOG categories on leading and lagging strands in low and high GSB species. Genes belonging to any 2 SuperCOG categories were considered in a separate group (SuperCOG 12, 13 and 23 groups). Values exceeding 2000 bp were omitted from the plots but were retained in the statistical analyses. In low GSB, n = 1842 in all categories except the following: SuperCOG 13-leading = 1841, SuperCOG 13-lagging = 1838. In high GSB, n = 526 in all categories except the following: SuperCOG 13-lagging = 493, SuperCOG 23-lagging = 522. Statistical significance was calculated using the Mann-Whitney *U*-test and *P*-values are presented on the plots. Gene length is expressed in base pairs (bp). Number of species is indicated by n**.**

The length of a gene is correlated with the function and complexity of the encoded product [4]. Further, the number and distribution of genes on leading and lagging strands are known to be driven by their essentiality and functionality [21,28]. Therefore, we examined whether these characteristics influence gene length in a strand-specific manner. For this, we obtained the gene essentiality information from the Database of Essential Genes [29] (DEG; 29 species). We then classified genes into essential and non-essential groups and compared their lengths across low and high GSB species. We observed that the mean length of essential genes is higher relative to non-essential genes in low and high GSB species, agreeing with previous observations [10]. In low GSB species, strand-specific gene length distributions were not significantly different, and in high GSB species, both essential and non-essential genes were shorter on the lagging strand (Fig 3A and 3B). We note that in high GSB species, a prominent difference of 57 bp was observed between the mean lengths of essential genes on the leading and lagging strands, albeit statistically insignificant (S1 Table). Interestingly, we also observed a greater median length of essential genes on the leading strand in high GSB species than in low GSB species, consistent with the strong leading strand bias of essential genes. Thus, the selective constraint on the lagging strand gene lengths is evident, even in the case of essential genes.

Since length is correlated to protein function, we categorized the genes based on broad functionality into protein-coding and non-coding across the bacterial domain. In both functional classes, reduced lagging strand gene lengths were evident, particularly in high GSB species (Fig 3C and 3D). We noted that in ~95% of species, the mean length of non-coding genes on the lagging strand was less than 250 bp, and the majority were ribosomal-associated or tRNA genes.

We further analyzed the influence of gene functionality on length by categorizing genes according to COG and SuperCOG classifications [30,31]. The orthology-based classification in COG analysis divides the genes into 26 categories, while the SuperCOG classification combines similar COG classes into 3 main groups for a comprehensive understanding [31] (SuperCOG 1 - cellular processes and signaling; SuperCOG 2 - metabolism; SuperCOG 3 - information storage and processing). Genes belonging to any 2 SuperCOG categories were considered in a separate group (SuperCOG 12, 13 and 23 groups) and those which are important for all processes were assigned under general function category. Notably, irrespective of the functionality, we found that lagging strand genes remained shorter in most of the groups (Fig 3E). In low GSB species, the exceptions were SuperCOG 3 and 12 groups, whereas in high GSB species, SuperCOG 1 was the only exception in which the length was similar between leading and lagging strands. We also analyzed the gene lengths in 25 COG categories (S category of unknown function was excluded). We found that uniformly, lagging strand genes were shorter in majority of the categories in both low and high GSB species (S5 and S6 Figs). We observed exceptions in 6 out of 25 categories (E, N, P, Q, U, and Y), in which gene length was similar between the strands or higher on the lagging strand, irrespective of GSB. Overall, genes being shorter on the lagging strand of DNA, irrespective of their essentiality or functionality, strongly suggests that strand-specific differences in gene length primarily arise from the dual nature of the replication machinery and its interference with transcription.

### Transcription units are shorter on the lagging strand

A hallmark of the bacterial genome is its organization of genes into operons. Operons are highly relevant and significant in the context of replication-transcription collisions, since all the genes in an operon are regulated by one or more promoters and transcribed as a single unit, thereby posing a longer hindrance to the replisome, especially on the lagging strand. Previous studies have observed that operons are preferentially located on the leading strand of replication, presumably to avoid the deleterious effects of head-on collisions between replication and transcription [16,25]. We therefore hypothesized that the selective constraint imposed by head-on RTCs would be stronger on the length of the transcription units (TUs) on the lagging strand.

**Fig 4:**
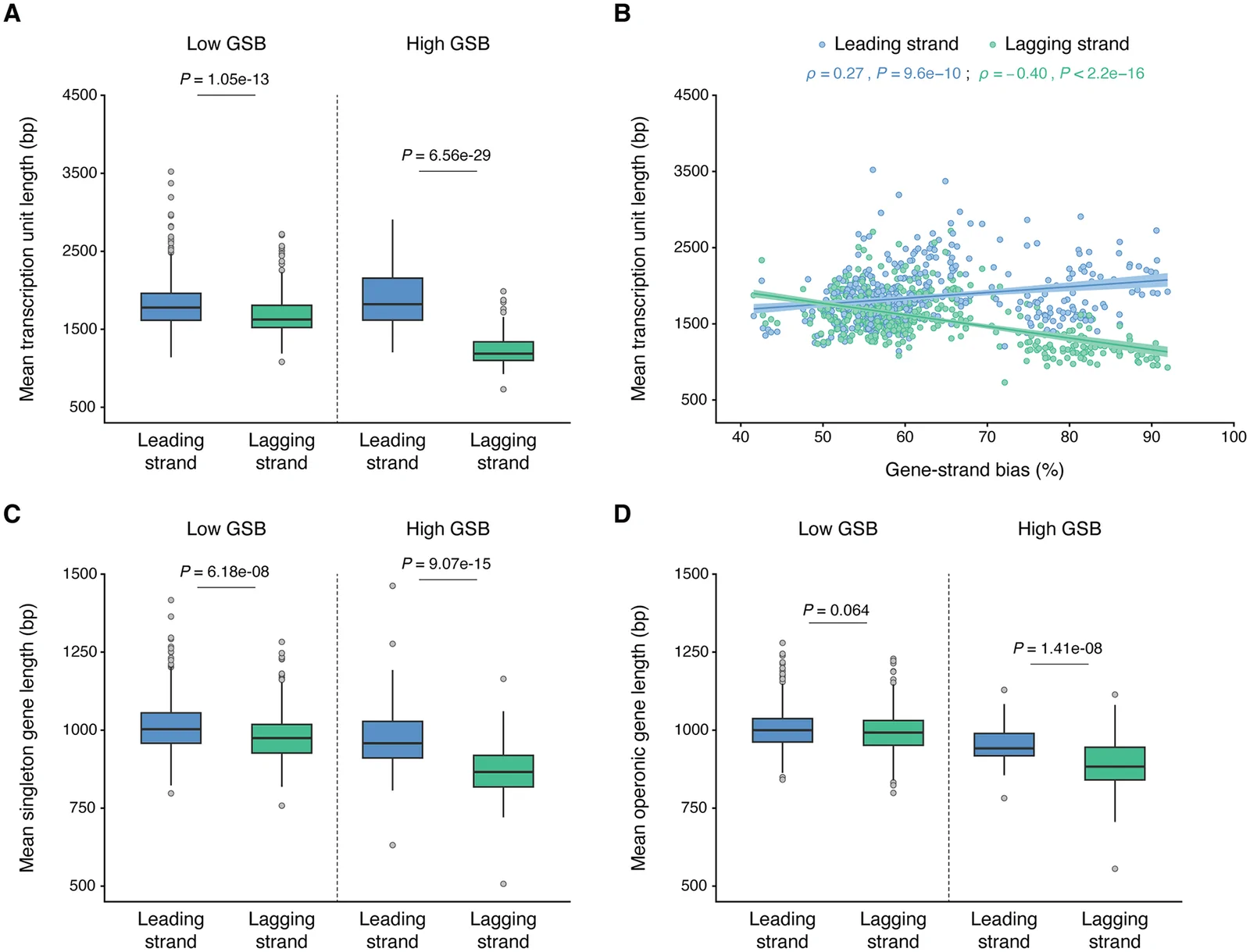
Transcription unit lengths are strongly constrained on the lagging strand. **(A)** Distribution of mean length of transcription units on leading and lagging strands in low (n = 396) and high (n = 100) GSB species. **(B)** Correlation between mean transcription unit length and GSB. Linear regression fits for leading and lagging strand transcription units are presented with 95% confidence intervals (496 species). Spearman’s rank correlation coefficients (*ρ*) and the *P*-values are presented on the plot. **(C)** Distribution of mean length of singleton genes on leading and lagging strands in low (n = 396) and high (n = 100) GSB species. **(D)** Distribution of mean length of operonic genes on leading and lagging strands in low (n = 396) and high (n = 100) GSB species. In **A**, **C**, and **D**, statistical significance was calculated using the Mann-Whitney *U*-test and *P*-values are presented on the plots. Length is expressed in base pairs (bp). Number of species is indicated by n**.**

We obtained the operon information for 496 species available in ProOpDB [32] and then classified genes into singleton and operonic transcription units. The distributions of mean lengths of transcription units on leading and lagging strands were significantly different, with a mean difference of 262 bp (S1 Table). Further, in both low and high GSB species, we observed significantly shorter lagging strand TUs, with a striking difference of 670 bp specifically in high GSB species (Fig 4A). The trend persisted even after accounting for species-specific differences (S7A and S7B Fig) and in PolC/non-PolC groups as well (S7C Fig). Thus, as with gene lengths, we found that strong selection pressure acts on transcription unit lengths on the lagging strand. Furthermore, we derived the association between the gradients of GSB and TU lengths. Unlike the inverse relationship observed for both leading and lagging strand gene lengths, we found that leading strand TU lengths are positively correlated, while the lagging strand TU lengths are negatively correlated with GSB (Fig 4B). The moderate positive correlation on the leading strand could suggest that the reduction in lagging strand TU length is offset by an increase in leading strand TU lengths. This is in line with an earlier study, wherein longer transcription units were proposed to be favored on the leading strand [15].

Since transcription units are comprised of singletons and operons, we analyzed the lengths of singleton genes and operonic genes separately. In line with our earlier observations, both categories had shorter genes on the lagging strand and greater differences in high GSB species (Fig 4C and 4D). We also found that a further reduction in lagging strand TU lengths was achieved by a smaller number of genes per operon (S8A and S8B Fig).

**Fig 5:**
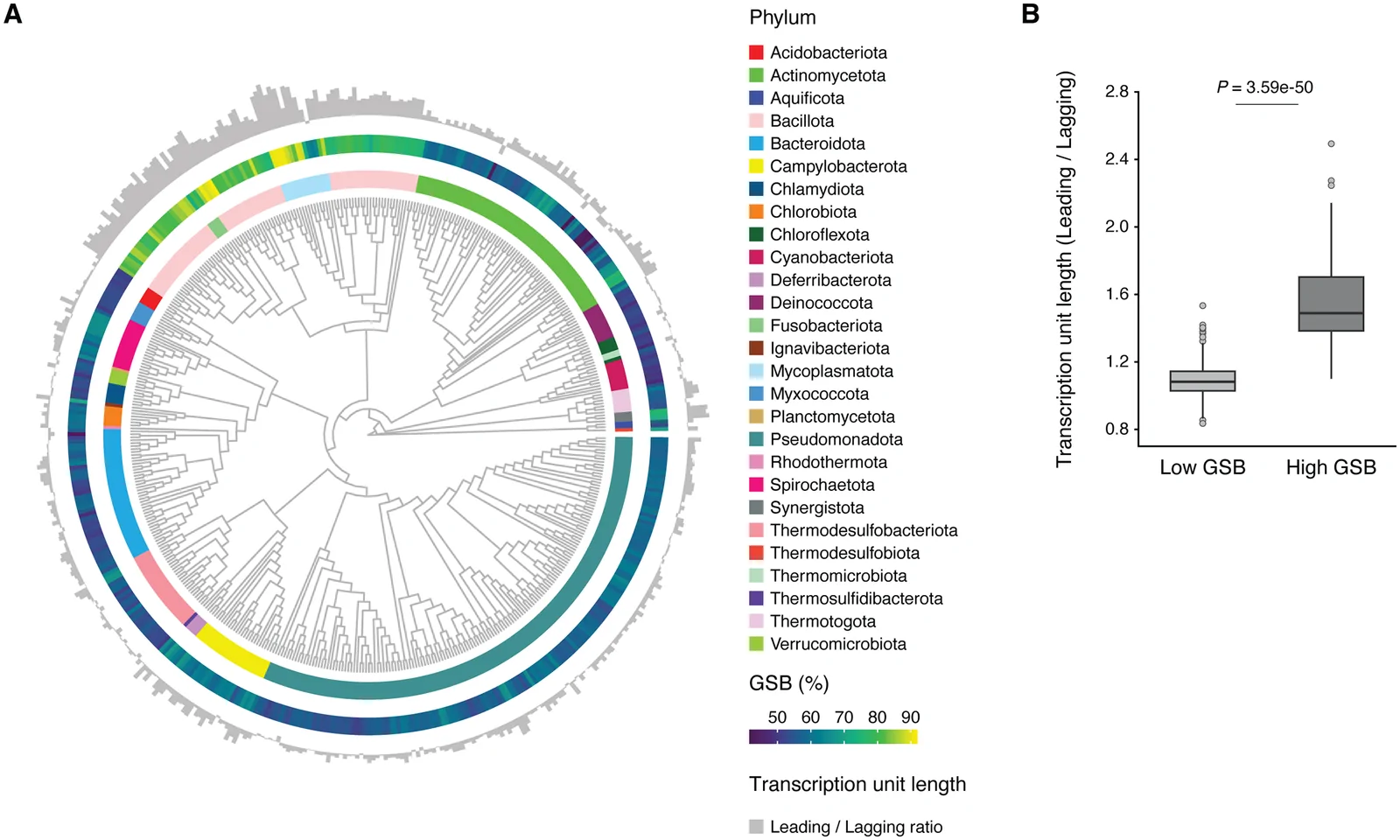
Transcription unit lengths are strongly constrained by nature of replication. **(A)** Phylogenetic summary of 496 species analyzed for transcription unit lengths. Phylum (1^st^), GSB (2^nd^), and the ratio of mean lengths of transcription units on the leading to the lagging strand (3^rd^) are plotted in concentric circles. The phylum information and the GSB scale are shown on the right. The ratio is plotted on a log scale and the bar heights correspond to the ratio. **(B)** Distribution of ratio of mean lengths of transcription units on leading and lagging strands in low GSB (n = 396) and high (n = 100) GSB species. Statistical significance was calculated using the Mann-Whitney *U*-test and *P*-value is presented on the plot. Number of species is indicated by n**.**

Finally, we mapped the TU length ratio and GSB of the 496 species on the phylogenetic tree and observed a striking concordance between high GSB and a greater leading-to-lagging ratio of transcription unit lengths (Fig 5A and 5B; S9 Fig). This clear association indicates strong selective constraints imposed on lagging strand transcription unit length, more stringently in species that have evolved to minimize the consequences of head-on replication-transcription collisions by achieving high GSB.

### Promoter nucleotide diversity increases with transcription unit length

Replication-transcription collisions affect genomic stability by causing replisome stalling, transcriptional interruption, R-loop accumulation, and spontaneous mutagenesis in bacteria [12–14,33,34]. The severity of RTCs is strand-dependent and is relatively more detrimental in the head-on orientation [26,27]. In bacteria, it has been reported that spontaneous mutation rates are higher in lagging strand transcription units with indels (insertions and deletions) and promoter base substitutions as the mutation signatures [14]. Specifically, the promoter base substitution was shown to occur predominantly due to head-on RTCs [14]. Promoter base substitutions are proposed to arise due to the susceptibility of the template strand to spontaneous DNA damage in the single-stranded state during transcription initiation. In particular, on the lagging strand, head-on RTCs can delay RNA polymerase promoter escape, leaving the promoter open complex in a single-stranded state for prolonged periods, thereby increasing DNA damage [14]. In line with this, it is conceivable that the longer the transcription unit on lagging strand, the greater the delay in promoter escape and the subsequent mutagenic DNA damage leading to higher promoter base substitution rates. To test this, we estimated the nucleotide diversity (a proxy for substitution rate) at the cis-regulatory intergenic regions of transcription units on leading and lagging strands in a selected set of species (n = 43) representing a range of GSB (~50 – 90%). To assess the impact of TU length on promoter base substitution rate, we compared the nucleotide diversity between TU length quartiles.

**Fig 6:**
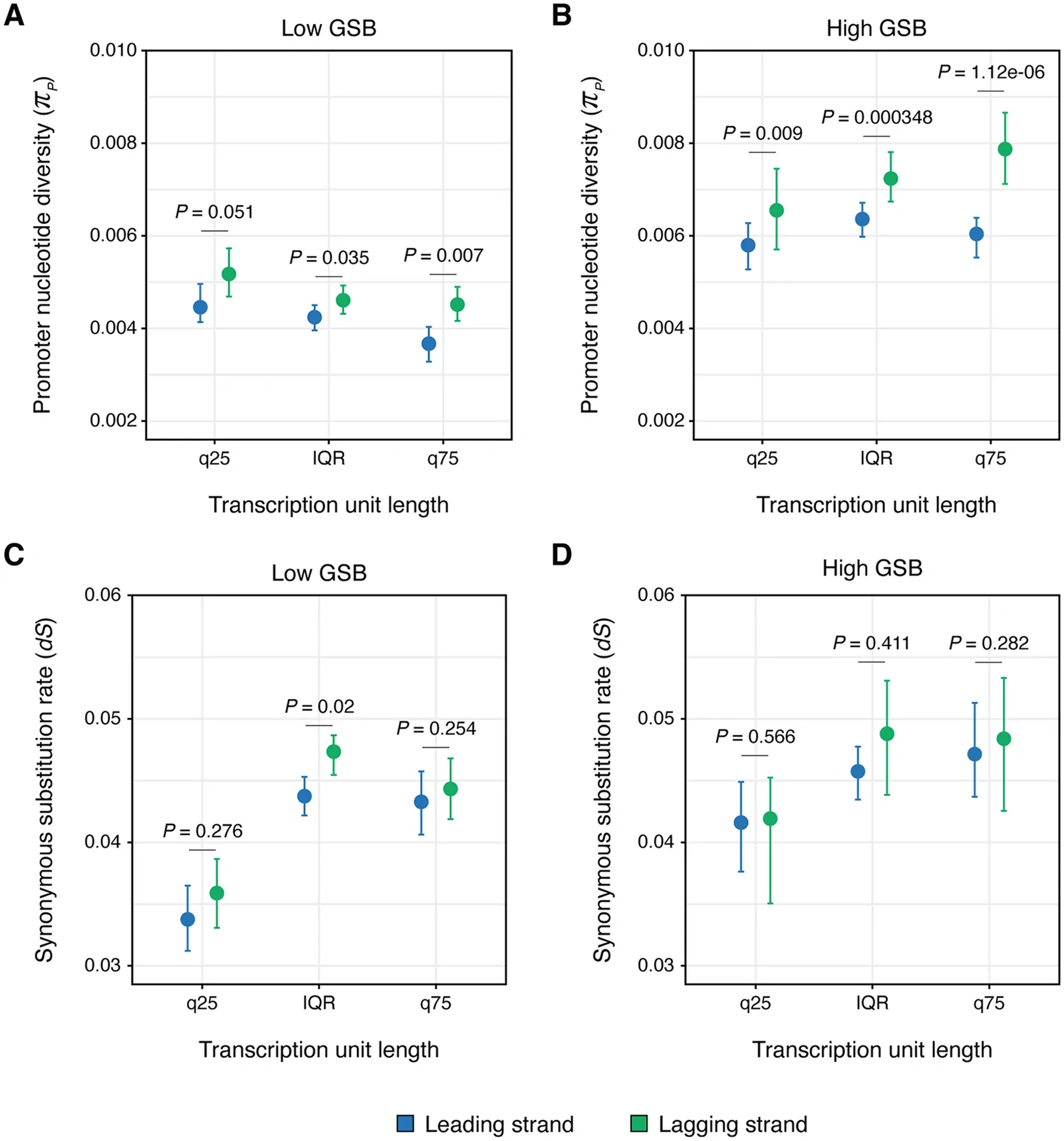
Transcription unit length-dependent increase in promoter base substitutions. **(A)** Nucleotide diversity (*π*_P_) in the promoters (intergenic regions) of leading and lagging strand transcription units in low GSB species plotted against the indicated quartiles of transcription unit length. **(B)** Same as in **A**, presented for high GSB species. **(C)** Substitution rate at the synonymous sites (*dS*), in the coding sequence (CDS) of leading and lagging strand transcription units in low GSB species against the indicated quartiles of transcription unit length. **(D)** Same as in **C**, presented for high GSB species. In **A**-**D**, medians with 95% confidence intervals are plotted for the distributions of mean values estimated from low GSB (23 species) and high GSB (20 species). Statistical significance was assessed using the Mann-Whitney *U*-test and *P*-values are presented on the plots.

We observed strand-specific differences in promoter nucleotide diversity (*π*_*P*_) across the length quartiles in both low and high GSB species, with the substitution rates consistently being higher on the lagging strand (Fig 6A and 6B). Strikingly, only the lagging strand transcription units, specifically in high GSB species, exhibited an increase in substitution rate with increasing TU length (Fig 6B). We examined whether the elevated substitution rates are caused by strand-specific differences in the length of intergenic regions (IGRs) encompassing promoters. We found that the mean length of IGRs was comparable between the leading and lagging strands and across low and high GSB groups, suggesting that it is the length of the transcription units that affects promoter substitution rate (S10A and S10B Fig). Further, to assess whether the length-dependent increase in substitution rate was specific to promoter base substitutions, we calculated the synonymous substitution rates (*dS*) in the coding regions. Synonymous sites are relatively less constrained by selection and nearly reflect the spontaneous mutation rates. We didn’t observe a strand-specific difference or length-dependent increase in the synonymous substitution rates in the coding region (Fig 6C and 6D). Therefore, the length-dependent effect specific to promoter base substitutions highlights that longer TUs are more vulnerable to promoter mutations. Taken together, we propose that the lengths of transcription units on the lagging strand are constrained by the selection pressure to minimize promoter base substitutions caused by head-on RTCs.

## Discussion

In this study, we identified a striking DNA replication strand-specific constraint imposed on the length of transcription units across the bacterial domain. We found that the genes and operons present on the lagging strand of replication are consistently shorter. The trend was prominent across all functional categories, indicating a universal selective constraint on gene lengths. We uncovered a crucial link between gene length and DNA replication machinery and its strand-specific conflict with the transcription complex. The length of transcription units specifically on the lagging strand decreases with increasing gene-strand bias (GSB) across bacterial species. Finally, we discovered a length-dependent increase in the base substitution rate in the promoter regions of lagging strand transcription units, suggesting replication- and transcription-dependent selection pressure acting on gene lengths in a strand-specific manner.

Earlier studies have established that the nature of replication machinery strongly influences the bacterial genome organization [20]. The asymmetric mode of DNA replication along the leading and lagging strands strongly underlies the strand-specific effects. In addition, the dichotomy in the composition of the bacterial replisome with a single or dual catalytic ⍺ subunits (DnaE or DnaE/PolC) distinctly affects both strands. These factors have been shown to differentially influence genomic features on leading and lagging strands as exemplified by gene-strand bias. We observed a strand-specific bias in restricting the length of the genes and operons on the lagging strand across bacterial species and thereby identified that a fundamental genic feature is controlled by DNA replication. This is likely to be true in eukaryotes also, as the mode of replication is similar to that of bacterial species that use two DNA polymerases [35].

Bacterial genome organization is also strongly influenced by replication-transcription collisions [22,25,36]. In particular, the deleterious effects of head-on RTCs on lagging strand are avoided or minimized by evolutionary and mechanistic measures: (i) preferential enrichment of genes on the leading strand to promote co-orientation with replication resulting in gene-strand bias, (ii) by specifically excluding a greater fraction of highly expressed genes on the lagging strand so that hinderance posed by frequent RNA polymerases is avoided and (iii) RTC-mitigating molecular players, including transcription factors and accessory helicases. Here, we propose that by reducing the transcription unit length on the lagging strand, the probability and severity of head-on RTCs are greatly reduced, since shorter TUs would shorten the duration of RNA polymerase occupancy on DNA and, consequently, mitigate the impediment to replication. Notably, we find that the constraint on lagging strand TU length is much stronger in species with PolC and high GSB, which may experience more detrimental effects due to head-on RTCs [20,22]. The severity of head-on collisions in these species is strongly reflected in the selection for co-orienting replication and transcription for the majority of the genome, especially essential genes [28]. RTCs are likely to be more severe in PolC species than in non-PolC species, as suggested by experimental observations and comparative genomic analysis. In PolC species such as *B. subtilis* and *L. lactis*, inversion of ribosomal operons causes greater sensitivity to head-on RTCs, whereas similar inversions are tolerated in non-PolC species such as *E. coli* [27,37,38]. Consistently, PolC species exhibit a genome-wide lower frequency of gene inversions than non-PolC species, with a median 25-fold difference [22]. Hence, the differential severity of RTCs between PolC and non-PolC species could stem from intrinsic differences in the replisome, combined with transcription processivity [25,39]. The differential severity of RTCs may also be attributed to the differences in RTC-mitigating factors [13]. Finally, RTCs are shown to cause higher rates of spontaneous mutations on the lagging strand compared to the leading strand in PolC species like *B. subtilis*, while no such strand-specific RTC-induced mutagenic effects are apparent in non-PolC species like *E. coli* [14,17,34,40].

Replication-transcription collisions have short-term fitness and long-term evolutionary consequences. RTCs cause replication and transcription stress, affecting cellular fitness. Notably, RTCs are an important source of spontaneous mutations and are known to cause indels (insertions and deletions) and promoter base substitutions in bacteria [14]. In particular, lagging strand promoters are highly vulnerable to mutations due to head-on RTCs, since the replication fork antagonizes transcription elongation, rendering the promoter open complex susceptible to DNA damage. Thus, longer TUs can exacerbate head-on RTCs and increase promoter DNA damage. Accordingly, in our analyses, we observed a length-dependent increase in the base substitutions in the promoter regions of lagging strand transcription units. Thus, restricting the length of transcription units on the lagging strand can be a robust protective mechanism to minimize cis-regulatory mutations. This is in striking contrast to the conventional notion that gene length primarily affects the mutability of coding sequences. We speculate that gene length could also be regulated by RTC-induced indel mutations. Since RTCs cause localized indels, especially in repetitive DNA, they can modulate gene lengths over evolutionary times, resulting in a confined range of gene lengths. In essence, the fundamental cellular processes that interact with DNA govern gene length and exert selection pressure to preserve the integrity of gene regulation in a strand-dependent manner.

## Materials and methods

### Bacterial genome data and source

Genome sequences (.fna), genome annotation (.gff3), and protein sequences (.faa) files were obtained from the NCBI Microbial Genomes database for the reference/representative species of the bacterial domain (https://www.ncbi.nlm.nih.gov/datasets/genome/). The list of representative/reference bacterial species was obtained from a previous study [22]. The dataset included 2368 genomes (S1 File), excluding *Clostridium butyricum* S-45–5 since its record is suppressed from NCBI RefSeq. Throughout the study, only chromosomes annotated as primary were analyzed. Our final dataset also included 10 archaeal genomes for rooting the phylogenetic trees and was not included in the gene length analysis. Various genomic features, including genome length, number of genes, replication origin/termination coordinates, and gene-strand bias (% genes on the leading strand), were obtained from the previous study [22] and re-verified for accuracy. The ncbi-taxonomist tool [41] was used to retrieve the taxonomic information.

### Determination of gene orientation

Genes were classified into leading and lagging strands following the method reported [22]. Briefly, the origin and terminus of replication were determined using a GC-skew-based method using custom Python scripts. The genomic coordinate with the lowest cumulative GC-skew was considered the origin of replication, and the one with the highest cumulative GC-skew was considered the replication terminus. The predicted origins and termini were also confirmed from the DoriC database for available species [42]. Using the origin and terminus of replication, the chromosome was divided into two replichores, and genes on the + strand on replichore 1 (origin to terminus) were classified as leading, and genes on the – strand of replichore 1 were classified as lagging, and vice versa for replichore 2 (terminus to origin). GSB was calculated as the percentage of genes on the leading strand for each species.

### Calculation of the length of genes

Gene lengths were calculated for all annotated genes from the start and end coordinates in the genome annotation files (.gff3) for each species (S1 File). Then, the mean and median gene lengths for the whole chromosome were determined, separately for the leading and lagging strands. To account for species-specific variation, the mean *z*-scores for leading and lagging strand gene lengths were determined. Bacterial species growth rate data were obtained from a previous study, from which we retrieved the growth rates for 453 species that overlapped with our data set [43–45]. The linearity or circularity of bacterial chromosomes was determined from genome annotation information (.gff3).

### Calculation of the length of transcription units

The ProOpDB database [32] of operon predictions was used to identify operonic and singleton genes, and we retrieved information for 496 species. Transcription units were identified by mapping predicted operons. We further curated the data with the following conditions to remove potentially inaccurate predictions: (i) all genes in an operon must be in the same gene orientation; (ii) intra-operonic intergenic length must not exceed 200 bp, and (iii) operon length does not exceed a quarter of the genome size. Any gene not belonging to an operon was considered a singleton. Operon lengths were calculated from the start coordinate of the first gene in the operon to the end coordinate of the last gene in the operon (S2 File).

### Classification of genes based on essentiality and functionality

Genes were classified into protein-coding and non-coding based on genome annotation in each species. Gene essentiality information was obtained from the DEG database [29], and we retrieved available information for 29 bacterial species. Essential genes from the DEG database were mapped to the reference species using BLASTN [v2.9.0+] [46] with the following cutoffs: identity ≥90%, query coverage ≥80%, and e-value ≤ 1e-05. In case of gene duplication, the gene with the best bit score was considered. All genes that were mapped from the DEG database were considered essential, while the remaining were categorized as non-essential. Species for which we were unable to map at least 50% of the essential genes were excluded.

To determine COG (Cluster of Orthologous Groups) [30] functional categories, gene ontology (GO) terms were extracted from genome annotation files for each coding sequence. The go2cog mapping tool [31] was then used to map the GO terms to the corresponding COG and SuperCOG categories. For genome annotations lacking GO terms, genomes were reannotated using the NCBI Prokaryotic Genome Annotation Pipeline (PGAP), and subsequently, COG categories were determined. Genes belonging to multiple COG categories were used for gene length calculations across all categories, whereas SuperCOG categories were mutually exclusive sets. The lengths of genes were calculated separately for the leading and lagging strands, for the following: (i) essential/non-essential; (ii) protein-coding/non-coding; (iii) COG and (iv) SuperCOG.

### Reconstruction of bacterial phylogeny, mapping, and visualization of genomic features

The phylogeny for the bacterial domain was obtained from the previous study [22], except for the excluded species *Clostridium butyricum* S-45–5 (GCF_003315755.1_ASM331575v1), which was pruned from the tree. Following the method described earlier, we reconstructed the phylogeny for the subset of species for which the transcription unit length analysis was performed (496 species; S2 File). The phylogenetic tree for the 496 species set was reconstructed from the marker gene alignment obtained from PhyloPhlan [47,48] using the IQTREE package [49] with the LG + F + R10 model and 1500 ultrafast bootstraps, with the archaeal species as the outgroup. For both trees, the outgroup species were removed before visualization. The ratios of gene lengths and TU lengths on the leading and lagging strands were mapped as continuous traits on the kingdom-wide phylogeny and the subset species dataset phylogeny, respectively, by reconstructing ancestral states using the fastAnc function in the phytools R package (1.5-1) [50]. The phylogenetic trees were visualized using the ggtree R package [51].

### Nucleotide diversity analysis

An in-house bioinformatic pipeline was developed for calculating nucleotide diversity in bacterial populations. The pipeline consists of a series of modules implemented in Python (v3.6 and above) as elaborated below.

#### Species selection for nucleotide diversity analysis.

We chose 43 species that span a broad range of gene-strand bias (~50–90%) and for which at least 10 strains representative of natural populations were available and used them to analyze nucleotide diversity in coding and intergenic regions. An unbiased phylogenetic approach was used to select the strains. For each species, a phylogenetic tree was constructed using PhyloPhlan [47,48] (v3.1.1, diversity = ‘low’, set to ‘fast’) from conserved protein sequences. Pairwise distances between strains were calculated using the fastDist function of the phytools R package (1.5-1) [50]. To ensure that nearly identical strains were not overrepresented and did not skew the analysis, we used only those strains with pairwise distances above a cutoff set to the maximum pairwise distance to select ~50 genomes. For species with fewer than 50 strains, all strains were used for the analysis.

#### Construction of core and pan genomes.

Core and pangenomes for each species were constructed for the nucleotide diversity analysis. Gene sequences were extracted using coordinates from the genomic.gff3 and genomic.fna file of the corresponding genome. An all-vs-all BLASTN (2.9.0+) was performed across all extracted genes, and genes with a reciprocal hit satisfying the cutoffs (identity ≥ 75%, length coverage ≥ 80%, e-value ≤ 1e-5) were used to construct the core and pan genomes excluding paralogs using a custom Python script.

#### Reconstruction of species phylogeny.

For each species, nucleotide and protein sequences of coding regions were extracted from cds_from_genomic.fna and protein.faa files, respectively. Only genes belonging to the strict core genome (>99%) were considered and aligned using the MAFFT program (v7.453) [52]. Codon-based nucleotide alignments were then generated using the PAL2NAL program (v14) [53]. Nucleotide alignments of all the core genes were then concatenated, and the species phylogeny was reconstructed using the maximum likelihood method implemented in the IQ-TREE package (v1.6.12) [49] with the nucleotide substitution model GTR + I + R and 1500 ultrafast bootstraps. An unrooted tree was used for all analyses.

#### Determination of gene orientation.

Genes were classified into leading and lagging strands as mentioned above, following the method reported [22].

#### Extraction of cis-regulatory intergenic regions.

Intergenic regions (IGRs) encompassing cis-regulatory elements were extracted from the reference genome using the annotation (.gff3) file’s genomic coordinates. IGRs with a length less than 50 bp or more than 300 bp are excluded from analysis. 15 bp upstream of the start codon was removed from the IGR because of the high probability of finding a Shine-Dalgarno sequence in that region. Also, 40% of the IGR downstream of the gene was removed due to the presence of transcription terminators in this region. Thus, the IGRs are likely to only contain potential promoters. To identify transcription unit promoters (singletons/operons), operons in each species were predicted using OperonMapper [54]. A promoter was considered operonic if the downstream gene was part of an operon in the reference genome; otherwise, it was considered a singleton. The lengths of the extracted promoter sequences were calculated, and the mean was obtained for each species. We excluded intergenic regions with bidirectional promoters since their lengths cannot be confidently determined.

#### Calculation of nucleotide diversity.

IGRs were aligned with MAFFT (v7.453) [52] using the ginsi method with --maxiterate 1000. Alignment quality was assessed using the GUIDANCE2 tool [55], and alignments with a median alignment score >0.9 were only considered. Nucleotide diversity (*π*), which serves as the proxy for mutation rate, was estimated using the nuc.div function in the R package Pegas (1.3) [56] for each promoter (*π*_*P*_). Synonymous substitution rates (*dS*) were estimated from the aligned nucleotide sequences of core genes (used earlier to reconstruct the phylogeny) using the CODEML program in PAML (4.9j) [57]. In the case of operons, the mean (*dS*) of all the CDS encoded in the operon was considered.

### Code, data visualization, and statistical analyses

Codes used in the study were written in Python (v3.6) unless otherwise noted. Plots were generated using the ggplot2 package in the R suite [58], and composite figures and schematics were generated using Adobe Illustrator (v27.7). Statistical analyses were performed in the R software suite (v4.5.2).

## Supporting information

S1 Fig Gene length varies depending on the DNA strand in bacteria irrespective of species differences. **(A)** Correlation between total coding sequence (CDS) length and total number of genes calculated from 2368 species representing the bacterial domain. Linear regression fit is represented by the blue line. Spearman’s rank correlation coefficient (*ρ*) and the *P*-value are presented on the plot. **(B)** Distribution of *z*-scores of mean gene lengths on leading and lagging strands determined from 2368 species. **(C)** Distribution of *z*-scores of mean gene lengths on leading and lagging strands in low (n = 1842) and high (n = 526) GSB species. In **C** and **D**, statistical significance was calculated using the Mann-Whitney *U*-test and *P*-values are presented on the plot. Number of species is indicated by n.
(PDF)

S2 Fig Genes are shorter on lagging strand in both slow and fast growing bacteria. **(A)** Distribution of mean gene lengths on leading and lagging strands in low (n = 341) and high (n = 42) GSB species which are slow growing. **(B)** Distribution of mean gene lengths on leading and lagging strands in low (n = 63) and high (n = 7) GSB species which are fast growing. Statistical significance was calculated using the Mann-Whitney *U*-test and *P*-values are shown on the plots. Number of species is indicated by n.
(PDF)

S3 Fig DNA strand-specific differences in gene length across species with linear chromosomes. **(A)** Distribution of mean length of genes on linear chromosomes between leading and lagging strands of DNA replication (146 species). **(B)** Distribution of mean gene lengths on linear chromosomes between leading and lagging strands in low (n = 130) and high (n = 16) GSB species. Statistical significance was calculated using the Mann-Whitney *U*-test and *P*-values are shown on the plots. Number of species is indicated by n.
(PDF)

S4 Fig Lagging strand genes are shorter across the bacterial domain. Bacterial domain phylogeny depicting the PolC status (1^st^ circle) and bacterial taxa (2^nd^ circle). The ratio of mean gene lengths of leading over lagging strands was treated as a continuous trait and mapped onto the branches by ancestral reconstruction. Each dashed line indicates the corresponding taxon (n = 2368 species). The absence/presence of PolC, phylum information and the gene length ratio scale are shown on the right. The bacterial domain phylogeny and its association with GSB is presented in Fig 2A.
(PDF)

S5 Fig Lagging strand genes are shorter across COG categories in low GSB species. Distributions of mean gene lengths for COG categories on leading and lagging strands in low GSB species. The labels for COG categories are depicted on the left. Number of species = 1842 in all categories except the following: D-lagging = 1793, E-leading = 1838, E-lagging = 1834, G-leading = 1841, I-lagging = 1841, K-lagging = 1841, N-leading = 1194, N-lagging = 1120, Q-leading = 1824, Q-lagging = 1812, T-lagging = 1841, U-leading = 1841, V-leading = 1814, V-lagging = 1797, W-leading = 1679, W-lagging = 1575, X-leading = 1610, X-lagging = 1485, Y-leading = 78, Y-lagging = 61, Z-leading = 844, Z-lagging = 585. Category S (Function unknown) was omitted from the analysis. Values exceeding 2000 bp were omitted from the plots but were retained in the statistical analyses. Statistical significance was calculated using the Mann-Whitney *U*-test and *P*-values are indicated on the plot.
(PDF)

S6 Fig Lagging strand genes are shorter in the majority of the COG categories in high GSB species. Distributions of mean gene lengths for COG categories on leading and lagging strands in high GSB species. The labels for COG categories are depicted on the left. Number of species = 526 in all categories except the following: A-lagging = 524, B-lagging = 525, D-lagging = 395, E-leading = 519, E-lagging = 497, G-lagging = 523, H-lagging = 523, I-lagging = 509, K-lagging = 518, L-lagging = 523, N-leading = 273, N-lagging = 180, O-lagging = 524, P-lagging = 525, Q-leading = 515, Q-lagging = 463, T-lagging = 520, U-lagging = 523, V-leading = 515, V-lagging = 420, W-leading = 463, W-lagging = 321, X-leading = 446, X-lagging = 358, Y-leading = 23, Y-lagging = 5, Z-leading = 253, Z-lagging = 19. Category S (Function unknown) was excluded from the analysis. Values exceeding 2000 bp were omitted from the plots but were retained in the statistical analyses. Statistical significance was calculated using the Mann-Whitney *U*-test and *P*-values are indicated on the plot.
(PDF)

S7 Fig Length of lagging strand transcription units is strongly constrained in high GSB and PolC species. **(A)** Distribution of *z*-scores of mean transcription unit lengths on leading and lagging strands of DNA replication (496 species). **(B)** Distribution of *z*-scores of mean transcription unit lengths on leading and lagging strands in low (n = 396) and high (n = 100) GSB species. **(C)** Distribution of mean length of transcription units on leading and lagging strands in species lacking (non-PolC, DnaE present; 405 species) or having PolC (DnaE and PolC present; 91 species). Statistical significance was calculated using the Mann-Whitney *U*-test and *P*-values are shown on the plots. Number of species is indicated by n.
(PDF)

S8 Fig Reduced number of genes on lagging strand operons. **(A)** Number of genes per operon encoded on the leading and lagging strands of DNA (496 species). **(B)** Number of genes per operon on leading and lagging strands in low (n = 396) and high (n = 100) GSB species. Statistical significance was calculated using the Mann-Whitney *U*-test and *P*-values are shown on the plots. Number of species is indicated by n.
(PDF)

S9 Fig Lagging strand transcription units are strongly constrained across the bacterial domain. Bacterial domain phylogeny depicting the PolC status (1^st^ circle) and bacterial taxa (2^nd^ circle). The ratio of mean transcription unit lengths of leading over lagging strands was considered as a continuous trait and mapped onto the branches using ancestral reconstruction. Each dashed line indicates the corresponding taxon (n = 496 species). The absence/presence of PolC, phylum information and the transcription unit length ratio scale are shown on the right. The association of transcription unit length with GSB is presented in Fig 5A.
(PDF)

S10 Fig Length of intergenic regions (IGRs) does not display strand-specific differences. **(A)** Distribution of mean length of IGRs on leading and lagging strands of DNA replication (43 species). **(B)** Distribution of mean IGR lengths on leading and lagging strands in low (n = 23) and high (n = 20) GSB species. Mean IGR lengths were calculated for species included in the nucleotide diversity analyses (Fig 6). Statistical significance was calculated using the Mann-Whitney *U*-test and *P*-values are shown on the plots. Number of species is indicated by n.
(PDF)

S1 Table Summary statistics of length of genes and transcription units in low and high GSB species. (PDF)

S2 Table Summary statistics of length of genes and transcription units in non-PolC and PolC species. (PDF)

S1 File Summary of bacterial domain gene length data. (CSV)

S2 File Summary of bacterial domain transcription unit length data. (CSV)

S3 File Nucleotide diversity data for promoter and CDS. (CSV)

S4 File Summary of essential and non-essential gene length data. (CSV)

S5 File Summary of COG and Super-COG classification gene length data. (CSV)

S6 File Ratio of leading and lagging strand gene lengths. (CSV)

S7 File Ratio of leading and lagging strand transcription unit lengths. (CSV)

S8 File Phylogenetic tree of the bacterial domain. (TXT)

S9 File Phylogenetic tree of the subset of bacterial species used for transcription unit analyses. (TXT)
