# Supplementary material for: Replication-transcription collisions impose DNA strand-specific constraints on gene length in bacteria
Source: PLoS Genet. 2026 Aug 24;22(8):e1012282. doi: 10.1371/journal.pgen.1012282 (PMC13521453; doi:10.1371/journal.pgen.1012282)
Supplement: Supplementary file 1 [file pgen.1012282.s001.pdf]

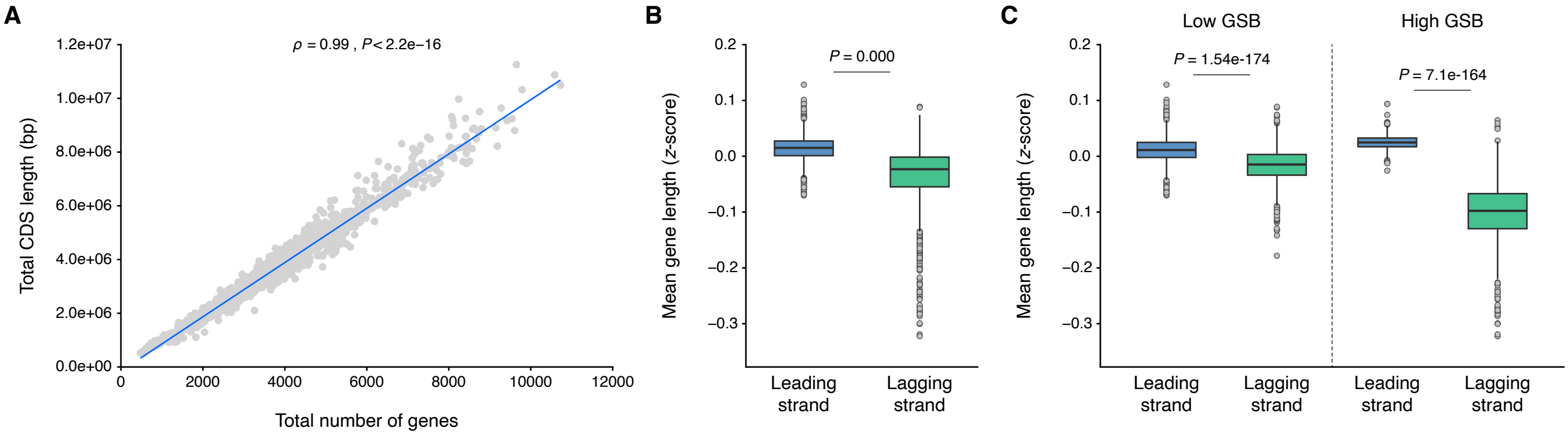

S1 Fig. Gene length varies depending on the DNA strand in bacteria irrespective of species differences. (A) Correlation between total coding sequence (CDS) length and total number of genes calculated from 2368 species representing the bacterial domain. Linear regression fit is represented by the blue line. Spearman's rank correlation coefficient (ρ) and the *P*-value are presented on the plot. **(B)** Distribution of *z*-scores of mean gene lengths on leading and lagging strands determined from 2368 species. **(C)** Distribution of *z*-scores of mean gene lengths on leading and lagging strands in low (n = 1842) and high (n = 526) GSB species. In **C** and **D**, statistical significance was calculated using the Mann-Whitney *U*-test and *P*-values are presented on the plot. Number of species is indicated by n.
