# Supplementary material for: Replication-transcription collisions impose DNA strand-specific constraints on gene length in bacteria
Source: PLoS Genet. 2026 Aug 24;22(8):e1012282. doi: 10.1371/journal.pgen.1012282 (PMC13521453; doi:10.1371/journal.pgen.1012282)
Supplement: Supplementary file 2 [file pgen.1012282.s002.pdf]

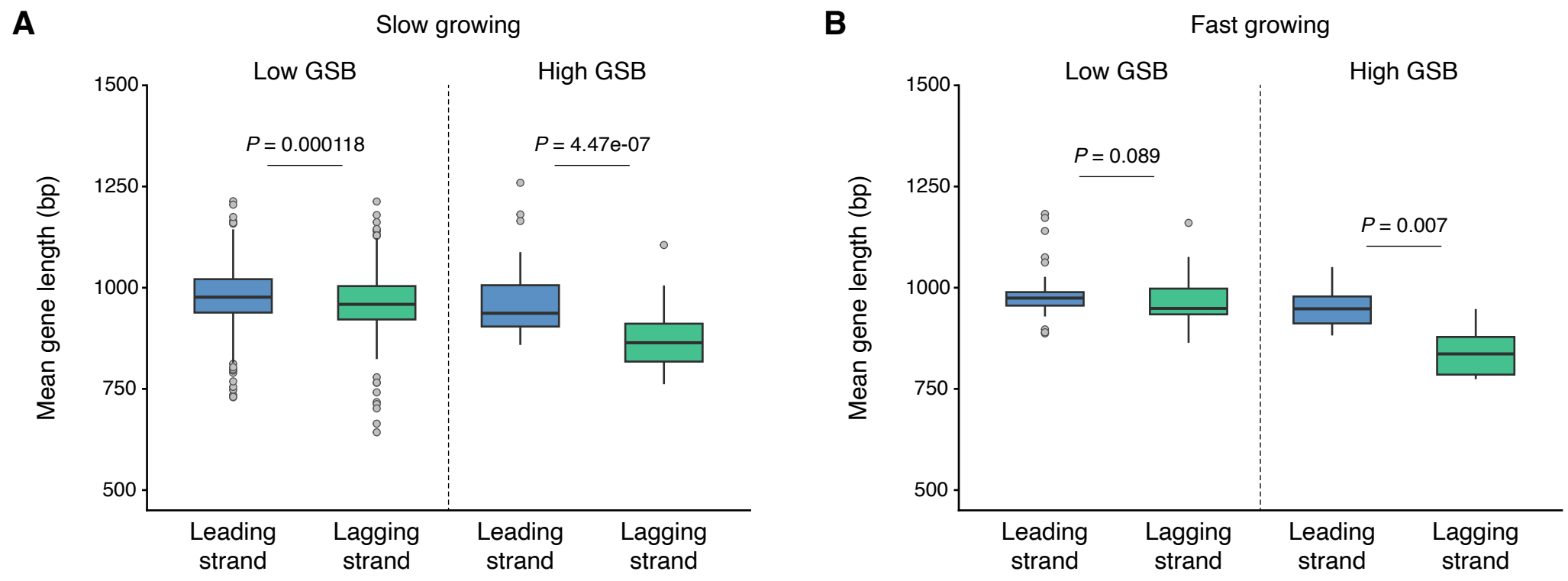

S2 Fig. Genes are shorter on lagging strand in both slow and fast growing bacteria. (A) Distribution of mean gene lengths on leading and lagging strands in low (n = 341) and high (n = 42) GSB species which are slow growing. **(B)** Distribution of mean gene lengths on leading and lagging strands in low (n = 63) and high (n = 7) GSB species which are fast growing. Statistical significance was calculated using the Mann-Whitney *U*-test and *P*-values are shown on the plots. Number of species is indicated by n.
