# Supplementary material for: Replication-transcription collisions impose DNA strand-specific constraints on gene length in bacteria
Source: PLoS Genet. 2026 Aug 24;22(8):e1012282. doi: 10.1371/journal.pgen.1012282 (PMC13521453; doi:10.1371/journal.pgen.1012282)
Supplement: Supplementary file 3 [file pgen.1012282.s003.pdf]

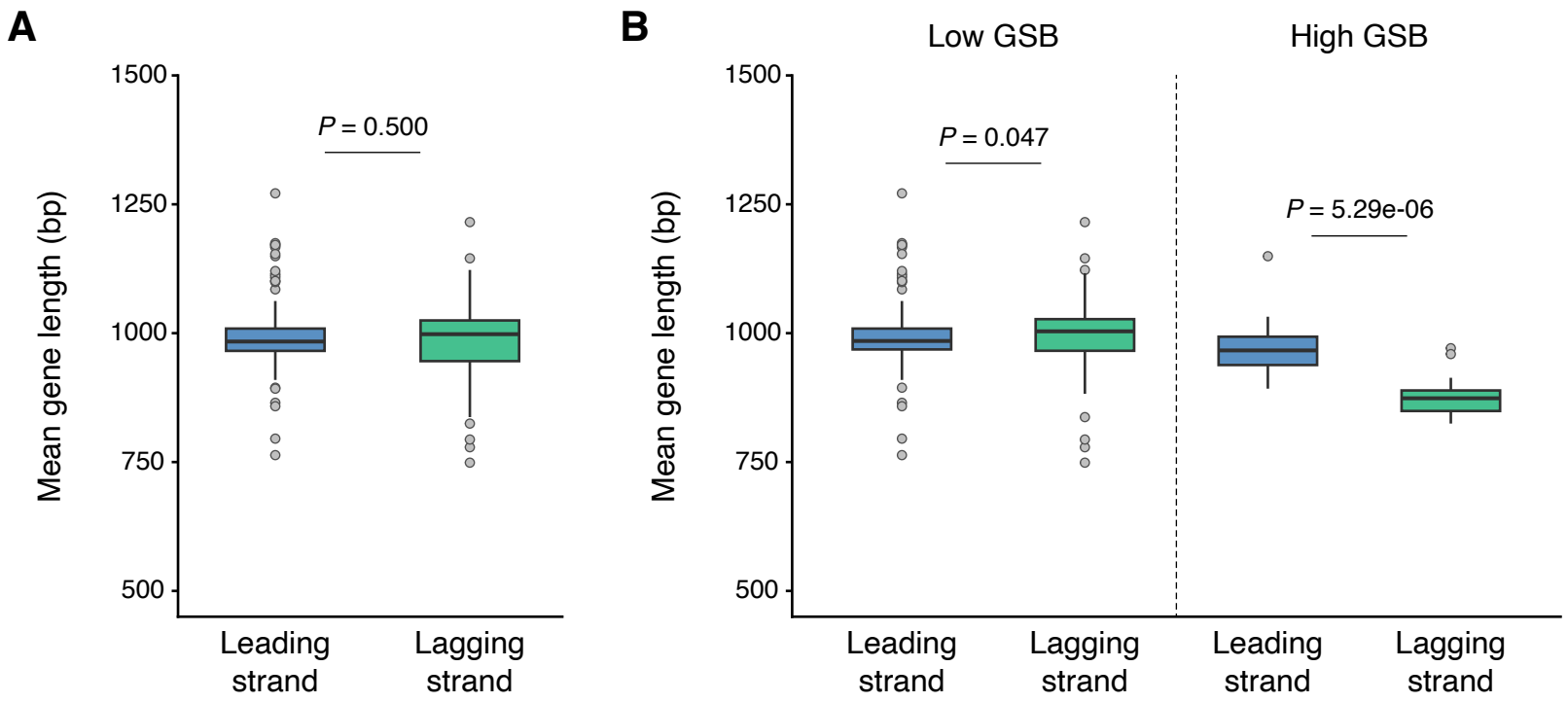

S3 Fig. DNA strand-specific differences in gene length across species with linear chromosomes. (A) Distribution of mean length of genes on linear chromosomes between leading and lagging strands of DNA replication (146 species). **(B)** Distribution of mean gene lengths on linear chromosomes between leading and lagging strands in low (n = 130) and high (n = 16) GSB species. Statistical significance was calculated using the Mann-Whitney *U*-test and *P*-values are shown on the plots. Number of species is indicated by n.
