# Supplementary material for: Replication-transcription collisions impose DNA strand-specific constraints on gene length in bacteria
Source: PLoS Genet. 2026 Aug 24;22(8):e1012282. doi: 10.1371/journal.pgen.1012282 (PMC13521453; doi:10.1371/journal.pgen.1012282)
Supplement: Supplementary file 4 [file pgen.1012282.s004.pdf]

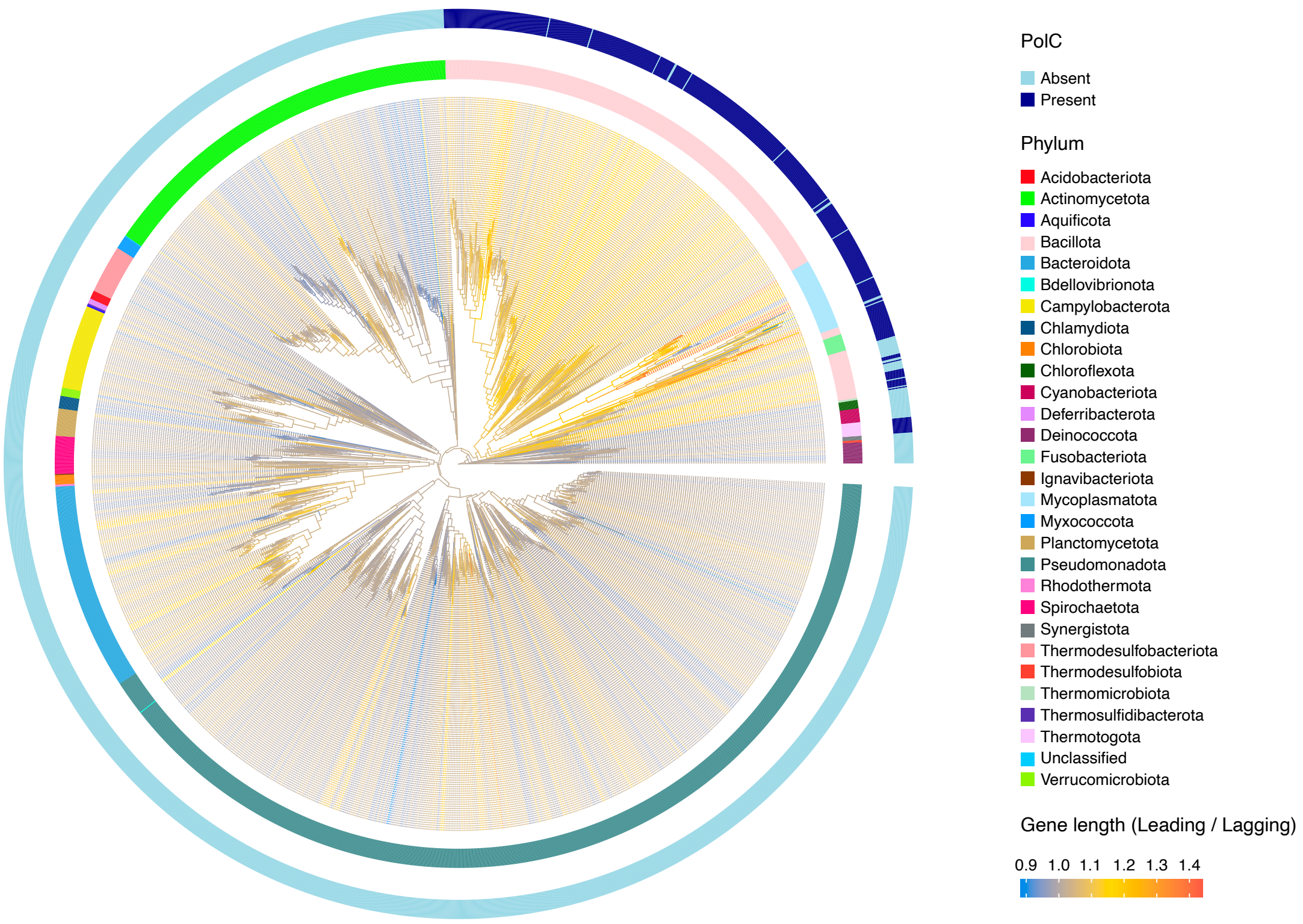

S4 Fig. Lagging strand genes are shorter across the bacterial domain. Bacterial domain phylogeny depicting the PolC status (1st circle) and bacterial taxa (2nd circle). The ratio of mean gene lengths of leading over lagging strands was treated as a continuous trait and mapped onto the branches by ancestral reconstruction. Each dashed line indicates the corresponding taxon (n = 2368 species). The absence/presence of PolC, phylum information and the gene length ratio scale are shown on the right. The bacterial domain phylogeny and its association with GSB is presented in Fig 2A.
