# Supplementary material for: Replication-transcription collisions impose DNA strand-specific constraints on gene length in bacteria
Source: PLoS Genet. 2026 Aug 24;22(8):e1012282. doi: 10.1371/journal.pgen.1012282 (PMC13521453; doi:10.1371/journal.pgen.1012282)
Supplement: Supplementary file 5 [file pgen.1012282.s005.pdf]

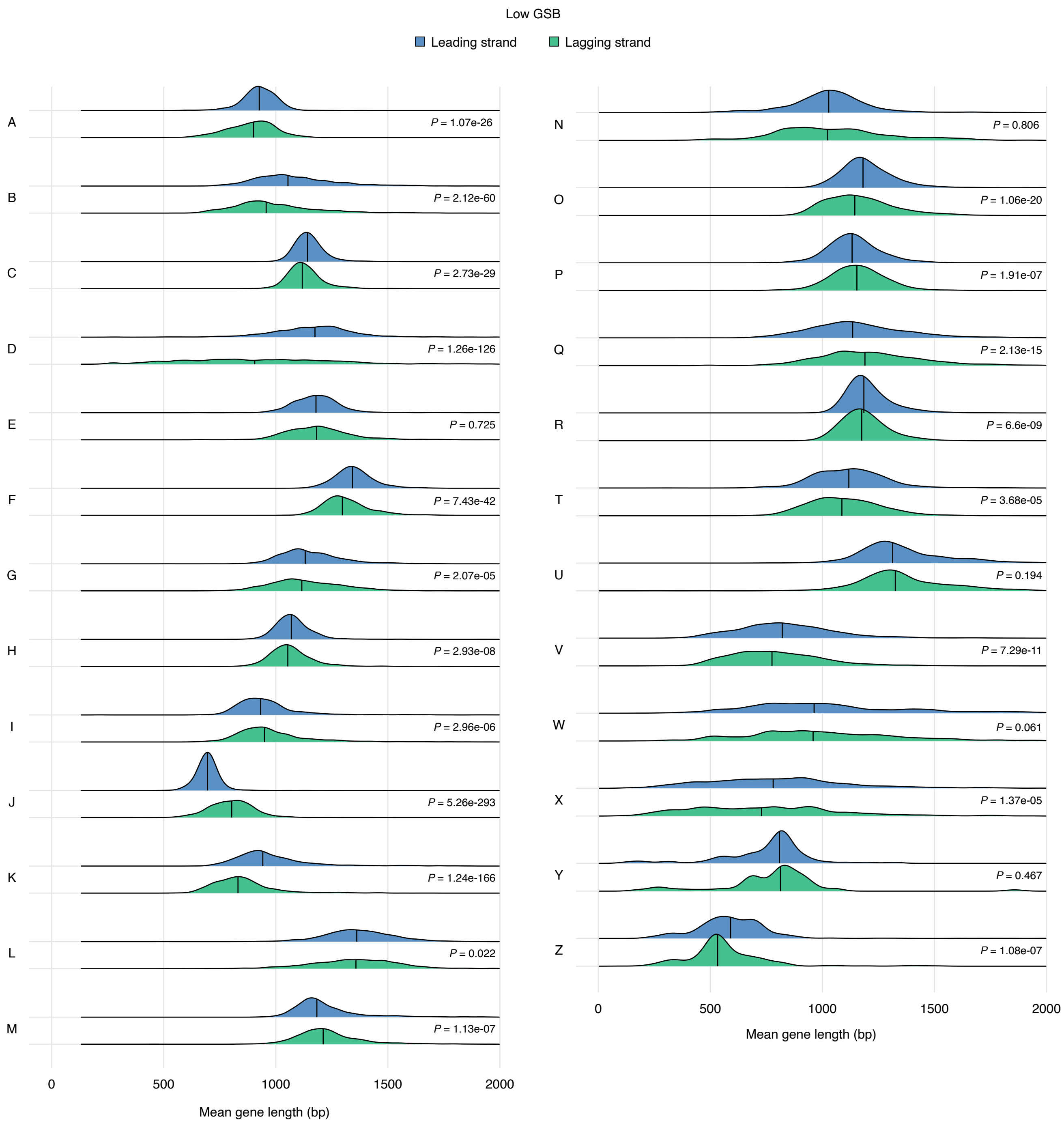

S5 Fig. Lagging strand genes are shorter across COG categories in low GSB species. Distributions of mean gene lengths for COG categories on leading and lagging strands in low GSB species. The labels for COG categories are depicted on the left. Number of species = 1842 in all categories except the following: D-lagging = 1793, E-leading = 1838, E-lagging = 1834, G-leading = 1841, I-lagging = 1841, K-lagging = 1841, N-leading = 1194, N-lagging = 1120, Q-leading = 1824, Q-lagging = 1812, T-lagging = 1841, U-leading = 1841, V-leading = 1814, V-lagging = 1797, W-leading = 1679, W-lagging = 1575, X-leading = 1610, X-lagging = 1485, Y-leading = 78, Y-lagging = 61, Z-leading = 844, Z-lagging = 585. Category S (Function unknown) was omitted from the analysis. Values exceeding 2000 bp were omitted from the plots but were retained in the statistical analyses. Statistical significance was calculated using the Mann-Whitney *U*-test and *P*-values are indicated on the plot.
