# Supplementary material for: Replication-transcription collisions impose DNA strand-specific constraints on gene length in bacteria
Source: PLoS Genet. 2026 Aug 24;22(8):e1012282. doi: 10.1371/journal.pgen.1012282 (PMC13521453; doi:10.1371/journal.pgen.1012282)
Supplement: Supplementary file 7 [file pgen.1012282.s007.pdf]

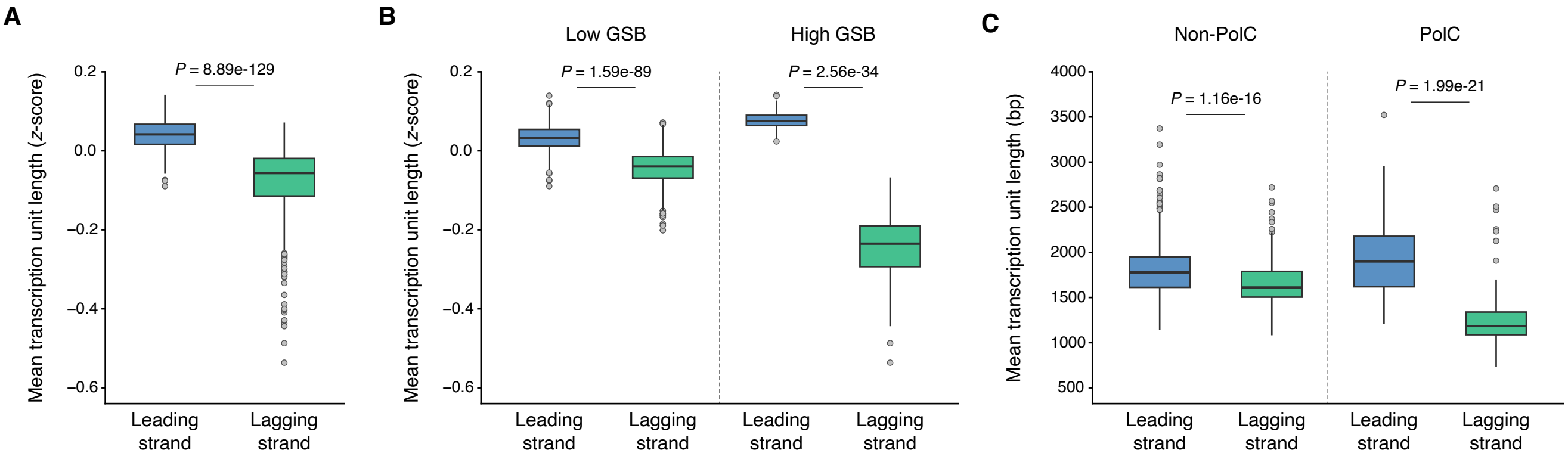

S7 Fig. Length of lagging strand transcription units is strongly constrained in high GSB and PolC species. (A) Distribution of *z*-scores of mean transcription unit lengths on leading and lagging strands of DNA replication (496 species). **(B)** Distribution of *z*-scores of mean transcription unit lengths on leading and lagging strands in low (n = 396) and high (n = 100) GSB species. **(C)** Distribution of mean length of transcription units on leading and lagging strands in species lacking (non-PolC, DnaE present; 405 species) or having PolC (DnaE and PolC present; 91 species). Statistical significance was calculated using the Mann-Whitney *U*-test and *P*-values are shown on the plots. Number of species is indicated by n.
