# Supplementary material for: Replication-transcription collisions impose DNA strand-specific constraints on gene length in bacteria
Source: PLoS Genet. 2026 Aug 24;22(8):e1012282. doi: 10.1371/journal.pgen.1012282 (PMC13521453; doi:10.1371/journal.pgen.1012282)
Supplement: Supplementary file 8 [file pgen.1012282.s008.pdf]

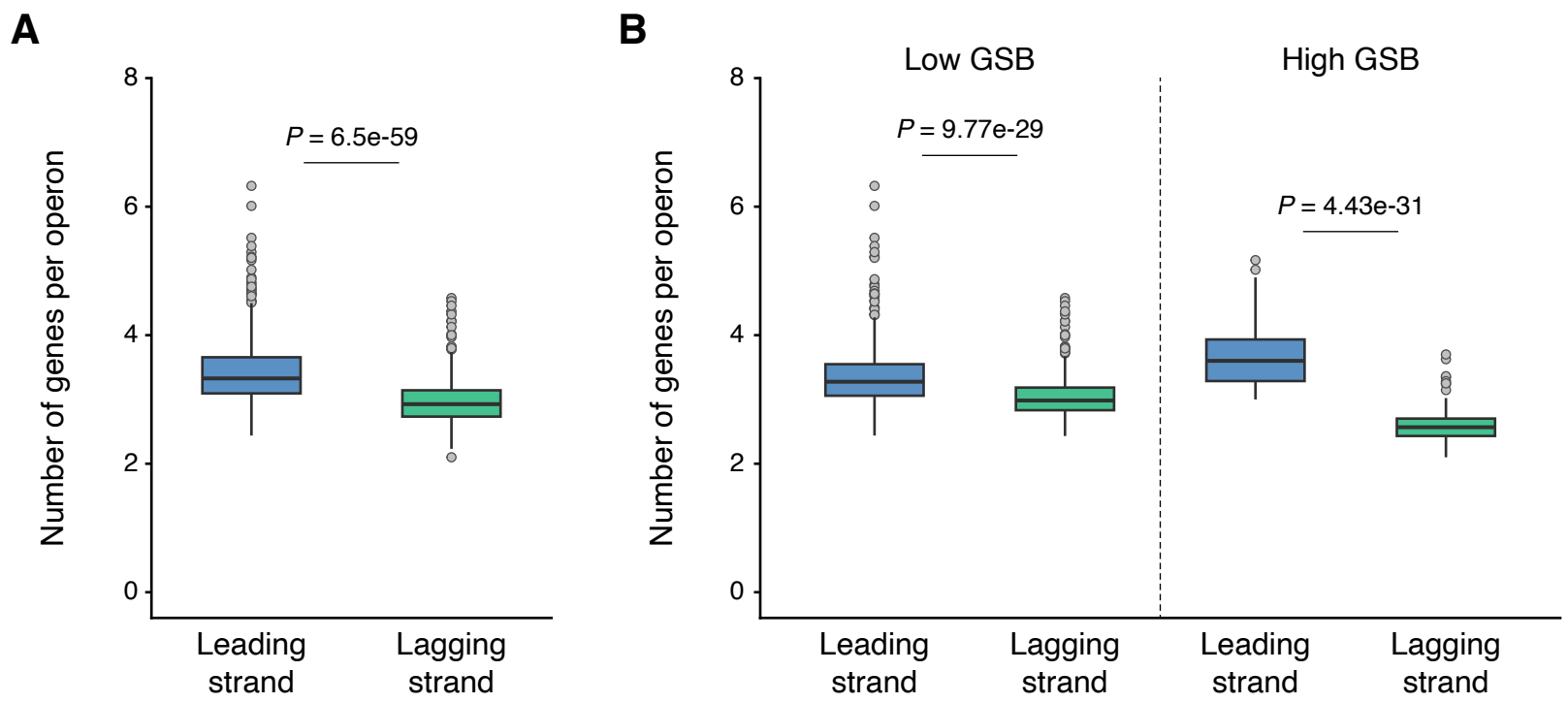

S8 Fig. Reduced number of genes on lagging strand operons. (A) Number of genes per operon encoded on the leading and lagging strands of DNA (496 species). **(B)** Number of genes per operon on leading and lagging strands in low (n = 396) and high (n = 100) GSB species. Statistical significance was calculated using the Mann-Whitney *U*-test and *P*-values are shown on the plots. Number of species is indicated by n.
