# Supplementary material for: Replication-transcription collisions impose DNA strand-specific constraints on gene length in bacteria
Source: PLoS Genet. 2026 Aug 24;22(8):e1012282. doi: 10.1371/journal.pgen.1012282 (PMC13521453; doi:10.1371/journal.pgen.1012282)
Supplement: Supplementary file 9 [file pgen.1012282.s009.pdf]

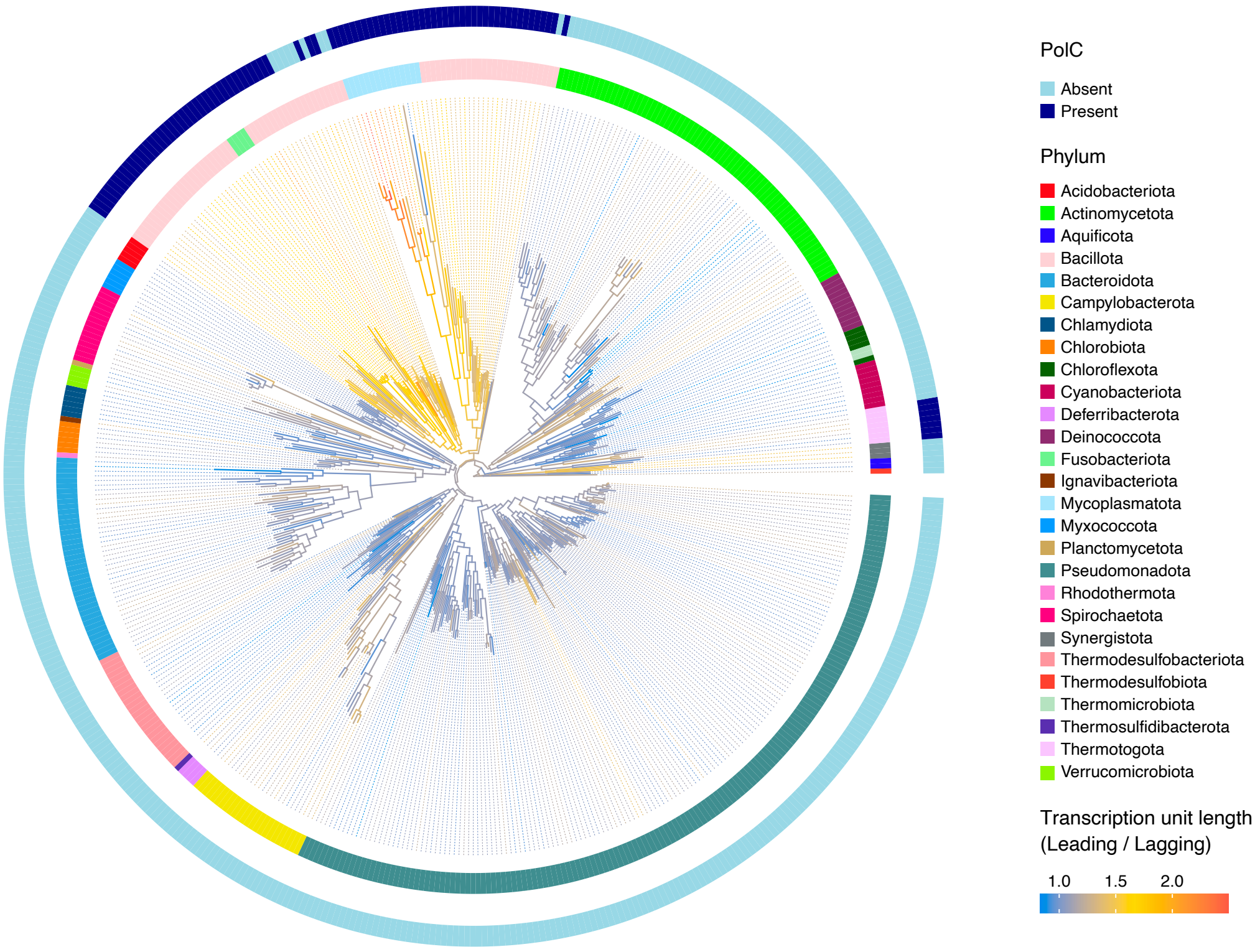

S9 Fig. Lagging strand transcription units are strongly constrained across the bacterial domain. Bacterial domain phylogeny depicting the PolC status (1st circle) and bacterial taxa (2nd circle). The ratio of mean transcription unit lengths of leading over lagging strands was considered as a continuous trait and mapped onto the branches using ancestral reconstruction. Each dashed line indicates the corresponding taxon (n = 496 species). The absence/presence of PolC, phylum information and the transcription unit length ratio scale are shown on the right. The association of transcription unit length with GSB is presented in Fig 5A.
