# Supplementary material for: Replication-transcription collisions impose DNA strand-specific constraints on gene length in bacteria
Source: PLoS Genet. 2026 Aug 24;22(8):e1012282. doi: 10.1371/journal.pgen.1012282 (PMC13521453; doi:10.1371/journal.pgen.1012282)
Supplement: Supplementary file 10 [file pgen.1012282.s010.pdf]

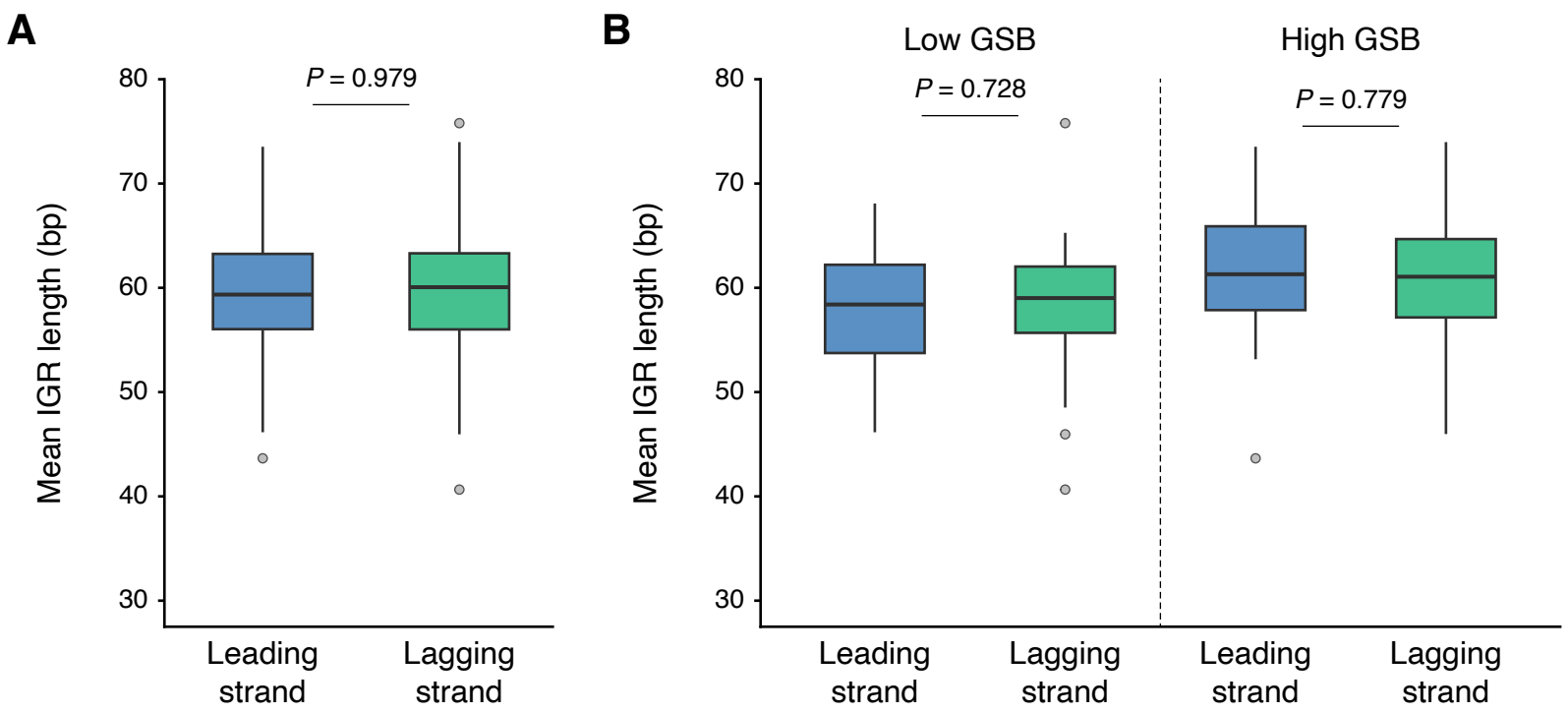

S10 Fig. Length of intergenic regions (IGRs) does not display strand-specific differences. (A) Distribution of mean length of IGRs on leading and lagging strands of DNA replication (43 species). **(B)** Distribution of mean IGR lengths on leading and lagging strands in low (n = 23) and high (n = 20) GSB species. Mean IGR lengths were calculated for species included in the nucleotide diversity analyses (Fig 6). Statistical significance was calculated using the Mann-Whitney *U*-test and *P*-values are shown on the plots. Number of species is indicated by n.
