# Supplementary material for: Replication-transcription collisions impose DNA strand-specific constraints on gene length in bacteria
Source: PLoS Genet. 2026 Aug 24;22(8):e1012282. doi: 10.1371/journal.pgen.1012282 (PMC13521453; doi:10.1371/journal.pgen.1012282)
Supplement: Supplementary file 11 [file pgen.1012282.s011.pdf]

S1 Table. Summary statistics of length of genes and transcription units in low and high GSB species.

Dataset			Mean (bp)	95%_CI_Mean (lower, upper)	Median (bp)	95%_CI_Median (lower, upper)
All genes	Low GSB	Leading	982.9	979.9, 985.8	976.3	973.8, 978.4
		Lagging	962.2	959.3, 965.1	955.3	952, 958.6
	High GSB	Leading	932.6	927.4, 937.8	924	918.8, 928.3
		Lagging	845.6	839.8, 851.4	843.9	836.6, 849.6
Essential genes	Low GSB	Leading	1049	962.2, 1135	1006	982, 1133
		Lagging	1059	958.9, 1158	1033	994.9, 1130
	High GSB	Leading	1038	959.5, 1117	1057	873.2, 1128
		Lagging	980.6	881.4, 1080	964.4	848.8, 1178
Non-essential genes	Low GSB	Leading	951	906.3, 995.7	948.4	897.4, 991.1
		Lagging	946.4	904.2, 988.6	924.5	895.2, 964.9
	High GSB	Leading	887.1	860.4, 913.8	886.8	836.8, 934
		Lagging	768.9	687.2, 850.7	793.1	602.5, 889.8
Protein-coding genes	Low GSB	Leading	998.9	995.9, 1002	992.5	989.7, 994.7
		Lagging	976.7	973.8, 979.7	969.8	966.8, 973.8
	High GSB	Leading	953.9	948.4, 959.4	945.8	942.1, 951.2
		Lagging	856.4	850.3, 862.5	853.5	847.3, 860.2
Non-coding genes	Low GSB	Leading	374.4	369.4, 379.3	381.9	375, 387.4
		Lagging	116.2	112.2, 120.3	93.58	93.04, 94.11
	High GSB	Leading	397.4	390.4, 404.4	402.1	391.2, 409.8
		Lagging	98.42	93.63, 103.2	80.67	79.67, 82.25
Transcription units	Low GSB	Leading	1837	1803, 1870	1779	1753, 1811
		Lagging	1678	1653, 1703	1625	1602, 1672
	High GSB	Leading	1902	1832, 1972	1822	1723, 1938
		Lagging	1232	1188, 1275	1187	1153, 1219
Singleton genes	Low GSB	Leading	1015	1006, 1024	1003	994.4, 1011
		Lagging	984	976.2, 991.8	974.2	965.8, 981.2
	High GSB	Leading	983.4	962.1, 1005	957.8	944.3, 1001
		Lagging	871.3	853.5, 889.2	865.8	853, 897.3
Operonic genes	Low GSB	Leading	1005	998.1, 1011	999.7	993.5, 1004
		Lagging	994.3	988, 1001	992	985.7, 998
	High GSB	Leading	953.9	942.1, 965.7	941.5	931.1, 956.3
		Lagging	894.3	877.6, 911	883.3	866.6, 912
