# Supplementary material for: Replication-transcription collisions impose DNA strand-specific constraints on gene length in bacteria
Source: PLoS Genet. 2026 Aug 24;22(8):e1012282. doi: 10.1371/journal.pgen.1012282 (PMC13521453; doi:10.1371/journal.pgen.1012282)
Supplement: Supplementary file 12 [file pgen.1012282.s012.pdf]

S2 Table. Summary statistics of length of genes and transcription units in non-PolC and PolC species.

**Dataset**			**Mean (bp)**	**95%_CI_Mean (lower, upper)**	**Median (bp)**	**95%_CI_Median (lower, upper)**
All genes	Non-PolC	Leading	981.2	978.3, 984.1	975.3	972.9, 977.8
		Lagging	959.7	956.7, 962.6	954.1	951, 958.0
	PolC	Leading	937.8	931.9, 943.7	924.6	920.2, 929.7
		Lagging	852.6	845.9, 859.3	846.9	839.5, 852.6
Transcription units	Non-PolC	Leading	1828	1796, 1859	1778	1751, 1809
		Lagging	1655	1632, 1679	1611	1595, 1648
	PolC	Leading	1948	1863, 2033	1899	1750, 2024
		Lagging	1289	1213, 1365	1184	1148, 1220
